# Influence of bisphenol A on growth and metabolism of *Vicia faba* ssp. minor seedlings depending on lighting conditions

**DOI:** 10.1038/s41598-022-24219-0

**Published:** 2022-11-24

**Authors:** Andrzej Kaźmierczak, Andrzej Kornaś, Małgorzata Mościpan, Justyna Łęcka

**Affiliations:** 1 Department of Cytophysiology, Institute of Experimental Biology, Faculty of Biology and Environmental Protection, University of Łódź, Pomorska 141/143, 90-236 Łódź, Poland; 2 Institute of Biology, Pedagogical University of Krakow, Podchorążych 2, 30-084 Kraków, Poland; 3 Institute of Heavy Organic Synthesis “Blachownia”, Energetyków 9, 47-225 Kędzierzyn-Koźle, Poland; 4 Laboratory of Environmental Threats, Department of Inorganic and Analytical Chemistry, Faculty of Chemistry, University of Łódź, Tamka 12, 91-403 Łódź, Poland

**Keywords:** Carbohydrates, Histocytochemistry, Proteins, Cell biology, Plant sciences, Analytical chemistry

## Abstract

The effect of one of anthropogenic pollutants, i.e., 4,4′-isopropylidenediphenol, called 2,2-bis (4-hydroxyphenyl) propane (BPA), at 30 and 120 mg L^−1^ concentrations in the darkness (DK) or dark/light (DK/LT) on growth and selected elements of metabolism of seedlings and leaf discs of *Vicia faba* ssp. *minor* was studied. Treatment with 120 mg L^−1^ BPA had greater effects which were reflected by increase in the number of necrotic changes in roots and stems as well as in leaf discs and reduction of the length of roots DK and DK/LT, and volume of roots in the DK group. However, minimal and no influence on the fresh and dry weight of roots and stems in plants growing under both types of lighting conditions were observed. In both DK and DK/LT groups these effects were correlated with reduced amounts of storage and cell wall-bound sugars as well as of proteins while in the DK/LT additionally with reduced soluble sugar levels in the roots and increased amounts of hydrogen peroxide and phenols in roots and stems as well as in treatment solutions, where these compounds were released. We suggest that endogenous phenols and BPA can be metabolised in roots and stems to quinones. It seems that TB-1,4-BQ, is the one of that of the five studied quinones. We expect that the results of this paper will help to answer the following question: does the phytomeliorative and phytosanitative *V. faba* ssp. *minor* plant is enough to be resistant on negative effects, and to be useful to reduce increasing amount of BPA in the environment?

## Introduction

Bisphenol A (BPA; 4,4′-isopropylidenediphenol or 2,2-bis (4-hydroxyphenyl) propane) is one of the best-known bisphenols and is important and frequently used in the manufacturing industry as a comonomer in the production of polycarbonate plastics and epoxy resins^1,2^.

BPA is present in electronic equipment (e.g., mobile phones, television sets, household appliances and others), items of daily use (e.g., medicines, reagents for scientific studies and liquid dishwashing detergent bottles, cosmetics jars), toys, food packaging and containers for beverages and water and easily migrates into the environment and is subsequently absorbed by humans through the gastrointestinal tract or skin^1,2^. Moreover, waste formed during production or resulting from the damage of the abovementioned items enters the environment, where it is absorbed from the soil and water by animals and plants and other uni- and multicellular organisms. In food webs, BPA circulates in the whole biosphere and also as microplastics^3^.

In several countries, including China, Norway, Canada, the United States, and Sweden, the production and sale of polycarbonate plastic baby bottles containing BPA has been banned^1^.

Even if BPA is no longer used, the amount of BPA in the environment will continue to increase^1,2^. BPA is a typical environmental oestrogen, which is referred to as a xenoestrogen, interfering with the endocrine system of wildlife and humans. Its trace amounts may damage human health by increasing the cancer rate, reducing immune function and impairing reproduction. BPA may be responsible for obesity in men. In the early 1930s, British biochemist Edward Charles Dodds showed that BPA mimicked oestradiol, but its effect was 37,000-fold weaker^4^. The toxic effects of BPA in animals, especially in mammals, is depend on its metabolites. Such as BPA diglucuronide, 5-hydroxy BPA isopropyl-hydroxyphenol, BPA glutathione conjugate, glutathionyl-phenol, glutathionyl 4-isopropylphenol, 4-methyl-2,4-bis(p-hydroxyphenyl)pent-1-ene (MBP) and BPA dimers.

BPA rapidly absorbed by plants via roots can be metabolised during hydroxylation and glucosylation or redox reactions into BPA mono-O-*β*-d-gentiobioside and the trisaccharide BPA mono-O-*β*-d-glucopyranosyl-(1 → 4)-[*β*-dglucopyranosyl-(1 → 6)] *β*-d-glucopyranoside, mono- and di-O-*β*-d-glucopyranosides and quinones. Then, BPA directly, or indirectly by products of its degradation, is introduced into human and animal bodies through fodder plants, vegetables or fruits as well as through tap water. Products of BPA degradation lose their estrogenic properties, but it does not mean that lose their toxic effects. Some of them show antibacterial, antifungal, parasitic and anticancer properties^5^.

The knowledge concerning the effects of BPA in animals is rather well documented^6^. However, in plants, data remain insufficient, especially regarding possible mechanisms of defence against its toxicity. Thus, it is important to know and understand the effects, which BPA can evoked in plants.

Studies with BPA have been performed on a limited scope and with a limited number of plant species, mainly with *Arabidopsis thaliana*^7^ and those of economic importance, i.e., soybean (*Glicine max*)^8^, lentil (*Lens culinaris*)^9^, tomato (*Lycopersicum esculentum*), lettuce (*Lactuca sativa*), soybean (*Glycine max*), maize (*Zea mays*) and rice (*Oryza sativa*)^10^ as well as with a plant used in water purification, i.e., *Lemna minor*^11^.

The results of research in plants showed that the effect of BPA depended on the BPA dose and stage of plant growth. The negative influence of BPA weakens with decreasing doses, as reflected in the positive changes observed in the successive growth stages of plants^2^, like in soybean seedlings where BPA at concentrations below 3 mg L^−1^ improved the growth^8^. Exactly, in *Arabidopsis thaliana,* BPA at 1 and 5 µM, regardless of dark, red, yellow, green, blue, and white light, caused increase in the fresh weight (FW), tap root length, and the lateral root formation but it had no notable role in germination and early seedlings growth. However, BPA dependent on the above mentioned light conditions influences the differentiation of the leaf blade^7^. In soybean seedlings BPA at 1.5 mg L^−1^ increased the plant height, FW and dry weight (DW) of stems and leaves and leaf area, while at higher concentrations, i.e., 7.0, 12.0, 17.2 and 50.0 mg L^−1^ the decrease in the growth parameters was observed^8^.

Some researchers suggest that the effects of BPA on cellular processes and the growth of plants may be related to its strong impact of plant hormone on gene expression, responsible for transcription. However, this impact remains poorly understood^12^. For example, in *A. thaliana*, BPA disrupts the auxin signalling and ethylene synthesis pathways, leading to a delay in flowering^13^. This effect was accompanied by increased indole-3-acetic acid (IAA) and zeatin (ZT) levels and decreased ratios of abscisic acid (ABA)/IAA, ABA/gibberellic acid (GA), ABA/ZT, ethylene (ETH)/GA, ETH/IAA, and ETH/ZT. The limiting effects of BPA on the growth of *A. thaliana* seedlings were observed at concentrations between 3 and 96 mg L^−1^, which were associated with reductions in IAA, GA, ZT, and ETH levels^10^.

The data related to BPA effects on plants presented in scientific literature are incomplete and incoherent; thus, general conclusions cannot be drawn. Thus, it seems that further studies are necessary. To address the above mentioned issues, the goal of the present research was to develop an experimental plan to study BPA effects on plant growth and development by analyses of its ability to (i) trigger necrotic changes (number of necrotic seedlings, range and area of necrosis and viability of cells); (ii) influence length, volume, FW and DW of roots and stems; (iii) change parameters of photosynthesis (the activity of PSII system and chlorophyll *a* [Chl *a*] fluorescence); (iv) influence metabolic parameters, i.e., the levels of phenols, hydrogen peroxides, quinones (QSs), the three most important types of sugars, and proteins and BPA levels in roots, stems and the culture solutions of plants cultured under darkness (DK) and darkness/light (DK/LT).

To reach the goal of the studies, 3-day-old seedlings of one of the most economically important Fabaceae fodder plants in Europe, namely, *Vicia faba* ssp. *minor*, were used. Its primary wild type variety *V. faba* ssp. *paucijuga* originates from Central Asia. *V. faba* ssp. *minor* is an annual plant with low soil and temperature requirements and is characterised by high nutritional value and harvesting potential.

Seeds provide exogenous amino acids, including lysine^14^, and many polyphenols^15^ necessary for the proper functioning of animal as well as plant organisms; thus, these components are the most important factors in this plant. *V. faba* ssp. *minor* develops symbiotic relationships, mainly with *Rhizobium* sp. Moreover, this plant improves the physical properties of soils through phytomelioration as well as phytosanitation^16^. Phytomeliorative properties of *V. faba* ssp. *minor* are related to the fact that its rapidly expanding root system facilitates the spread of respiratory gases and nitrogen compounds in the soil, reducing the need for artificial fertilisation. Moreover, this symbiosis stimulates the development of soil actinomycete microorganisms, such as *Streptomyces*, which additionally enrich the soil with nitrogen. In addition, these microorganisms produce bioactive compounds that help to limit the spread of weeds and crop plant diseases caused by fungal and bacterial pathogens^12,14^.

Based on the above mentioned properties, *V. faba* ssp. *minor* has been used as an experimental model in the Department of Cytophysiology at the Faculty of Biology and Environmental Protection University of Łódź (Poland) for research on plant morphology as well as biochemistry and signal transduction pathways and epigenetics for many years^17–19^.

Key words of the paper and List of Abbreviations used in the paper are presented in Supplementary Table S1.

## Results

### The number of necrotic seedlings, chlorophyll fluorescence, photosynthetic activities and necrotic areas in leaf discs

Macroscopic analyses showed that BPA damaged seedlings under both DK (Supplementary Fig. S1A) and DK/LT (Supplementary Fig. S1B) conditions.

**Figure 1:**
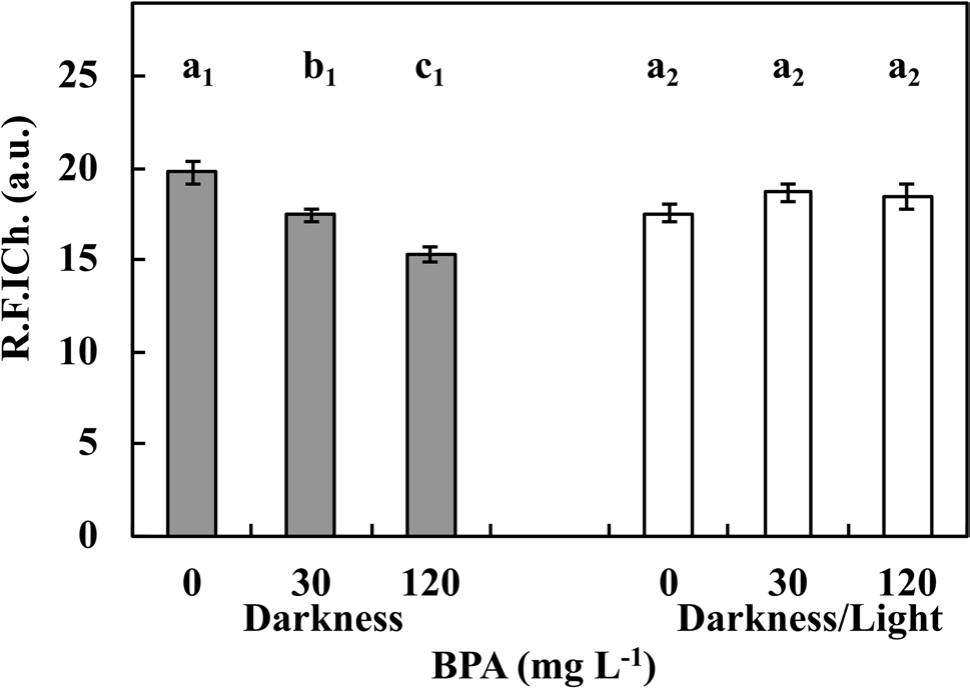
Red fluorescence intensity of chlorophyll (R.F.I.Ch.) in 1-cm-leaf discs of 2-month-old *V. faba* ssp. *minor* cultured for 72 h in the darkness or in the darkness/light under Ctrl (0 mg BPA) conditions or with 30 mg and 120 mg L^−1^ BPA. Bars indicate ± SE of the results from three biological replicates. Identically labelled columns indicate results that are not significantly different at *p* ≤ 0.05 for darkness and darkness/light groups, separately.

Chl fluorescence (R.F.I.Ch.) in the leaf discs of Ctrl cultivated for 72 h in DK (Supplementary Fig. S2A) and DK/LT (Supplementary Fig. S2D) measured using the images (Supplementary Fig. S2A′,D′) under blue LT were approximately 200 a.u. (Fig. 1). Under the same conditions, this parameter was reduced (*p* ≤ 0.05) by approximately 12.5% and 25% (Fig. 1) with 30 mg L^−1^ BPA (Supplementary Fig. S2B′) and 120 mg L^−1^ BPA (Supplementary Fig. S2C′) treatment, respectively. However, in DK/LT (Supplementary Fig. S2E′,F′), the R.F.I.Ch. values were similar (*p* > 0.05) (average 175 a.u.) compared to the Ctrl (Fig. 1).

**Figure 2:**
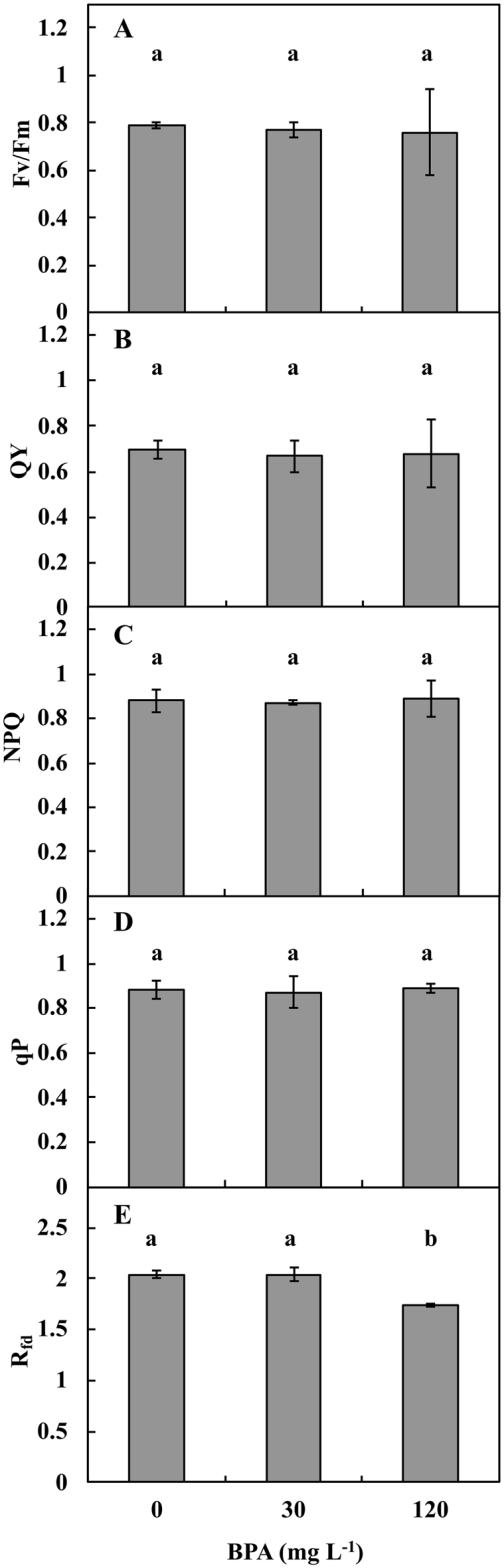
The values of chlorophyll *a* (photosystem II, PSII) fluorescence parameter of *V*. *faba* ssp. *minor* seedlings cultured under Ctrl (0 mg BPA) conditions or with 30 or 120 mg L^−1^ BPA darkness/light for 72 h. Ratio of variable to maximum fluorescence—the quantum efficiency of open PSII centers, (Fv/Fm, **A**); maximum fluorescence yield, quantum yield in light-adapted steady-state, (Fm); steady-state PSII quantum yield in light (QY, **B**); steady-state non-photochemical quenching in light, (NPQ, **C**); photochemical quenching, (qP, **D**); empiric parameter used to assess plant vitality (R_fd_, **E**). Identically labelled columns indicate results that are not significantly different at *p* ≤ 0.05.

In the stems in the DK/LT in Ctrl, 30 and 120 mg L^−1^ BPA, the values obtained for F_v_/F_m_ PSII quantum yield (Fig. 2A), steady-state PSII quantum yield (QY; Fig. 2B), steady-state nonphotochemical quenching (NPQ; Fig. 2C) and photochemical quenching (qP; Fig. 2D) were similar. Only the empirical parameter, i.e., R_fd_, used to assess plant vitality differed (Fig. 2E). There were similar R_fd_ values for 30 mg L^−1^ BPA and Ctrl and reduced in 120 mg L^−1^ BPA (*p* ≤ 0.05) by approximately 25% (*p* ≤ 0.05).

**Figure 3:**
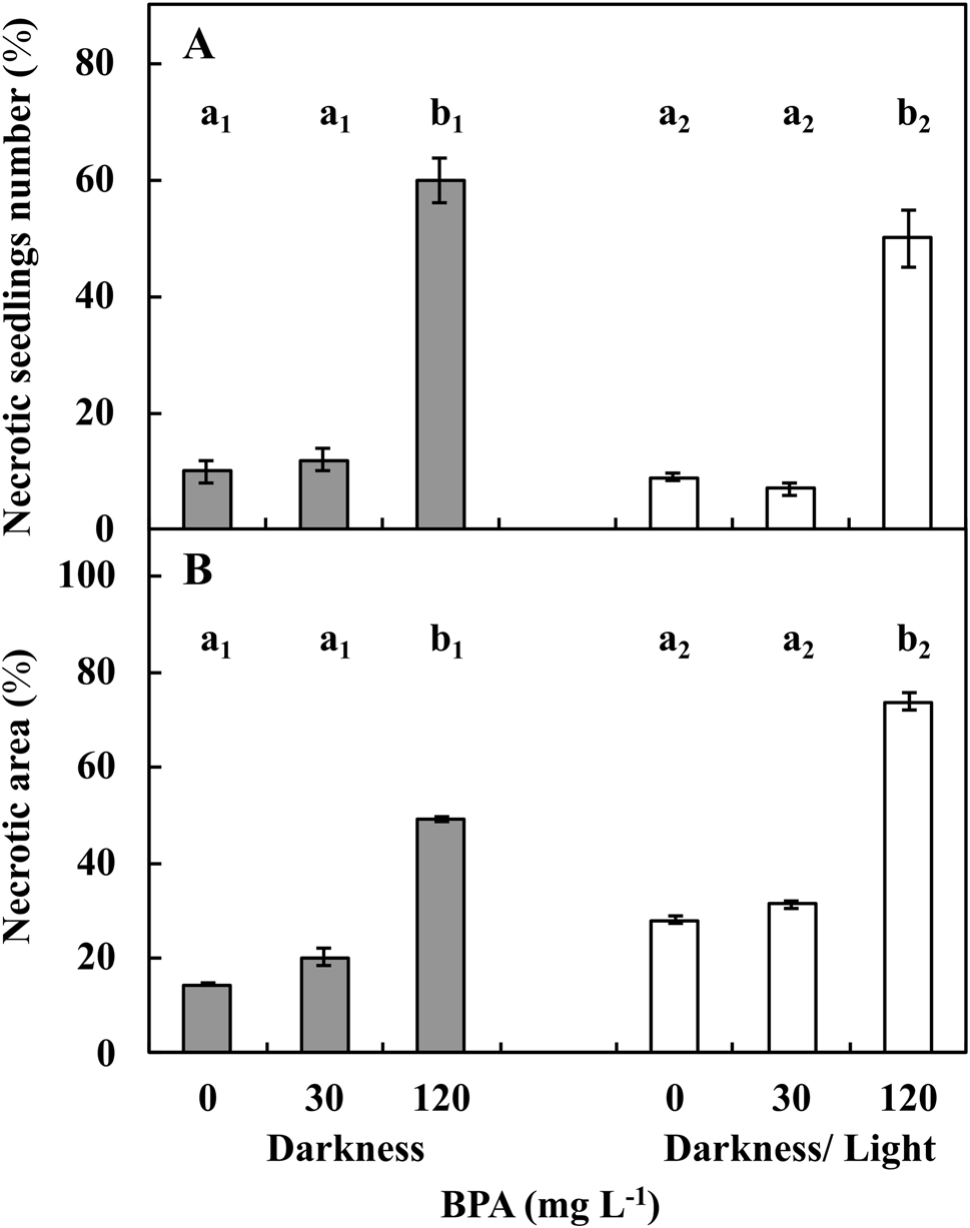
Percentage of necrotic seedlings (**A**) and necrotic areas in leaf discs of 2-month-old plants (**B**) of *V*. *faba* ssp. *minor* cultured for 72 h in darkness or darkness/light under Ctrl (0 mg BPA) conditions or with 30 or 120 mg L^−1^ BPA. Identically labelled columns indicate results that are not significantly different at *p* ≤ 0.05 for darkness and darkness/light groups, separately.

Upon treatment with 30 mg L^−1^ BPA, 13% and 7% of seedlings under DK and DK/LT conditions, respectively, were necrotic, similar results were observed in the Ctrl group (9% and 10%, respectively). Upon treatment with 120 mg L^−1^ BPA, the number of necrotic seedlings was on average six and fivefold increased (*p* ≤ 0.05) under DK and DK/LT conditions, respectively, compared to both Ctrl and 30 mg L^−1^ BPA (Fig. 3A). Thus, in the 120 mg L^−1^ BPA series, necrotic areas were identified in the arbitrarily distinguished apical, subapical, subbasal and basal zones of stems (Supplementary Fig. S3A, Is′–IVs′; Supplementary Fig. S4A, Is′–IVs′) and roots (Supplementary Fig. S3A, Ir′–IVr′; Supplementary Fig. S4A, Ir′–IVr′) of seedlings compared with the Ctrl series (Supplementary Fig. S3A, Is–IVs and Supplementary Fig. S4A, Is–IVs; Supplementary Fig. S4A, Ir–IVr and Supplementary Fig. S4A, Ir–IVr, respectively). Under both DK (Supplementary Fig. S3) and DK/LT (Supplementary Fig. S4) conditions, dark spots on stems (Supplementary Fig. S4A, Is′–IIIs′; Supplementary Fig. S4A, Is′, IIs′ and IVs′) and roots (Supplementary Fig. S3A, IIr′, IIIr′; Supplementary Fig. S4A, Ir′, IIIr′, IVr′) and injuries resembling mechanical damage (Supplementary Fig. S3A, IVs′ and Supplementary Fig. S4A, IIIs′; Supplementary Fig. S3A, Ir′, IVr′; Supplementary Fig. S4A, IIr′) were observed (*p* ≤ 0.05).

Necrotic changes were noted in approximately 30% and 20% (Fig. 3B) area of the leaf discs (Supplementary Fig. S2A–F) in the DK (Supplementary Fig. S2B; Fig. 3B) and DK/LT (Supplementary Fig. S2E; Fig. 3B), groups respectively. These levels were increased (*p* ≤ 0.05) by approximately 28% and 14%, respectively, upon 30 mg L^−1^ BPA treatment compared with Ctrl (Supplementary Fig. S2A; Fig. 3B). Treatment with 120 mg L^−1^ BPA enhanced the necrotic areas by approximately 40 and 240% (*p* ≤ 0.05) in the DK (Supplementary Fig. S2C) and DK/LT (Supplementary Fig. S2F) groups, respectively, compared to Ctrl.

### Cell viability in roots and stems of seedlings

Analyses of the viability of particular cells in roots and stems revealed three different cell populations (Supplementary Fig. S5a–f): cells with green nuclei, representing 95% living cells (Supplementary Fig. S5A,B); cells with orange-red nuclei, representing dying-dead cells and dead cells with damaged and fragmented nuclei (Supplementary Fig. S5C,D); and cells without nuclei (Supplementary Fig. S5E,F). The latter population was observed in the apical, subapical, subbasal and basal necrotic zones in roots and stems (Supplementary Figs. S1A,B; S3 and S4). The observations also showed that the living and necrotic regions in roots and stems were clearly separated (Supplementary Fig. S5F).

### Length, volume, fresh and dry mass of root and stem seedlings

**Figure 4:**
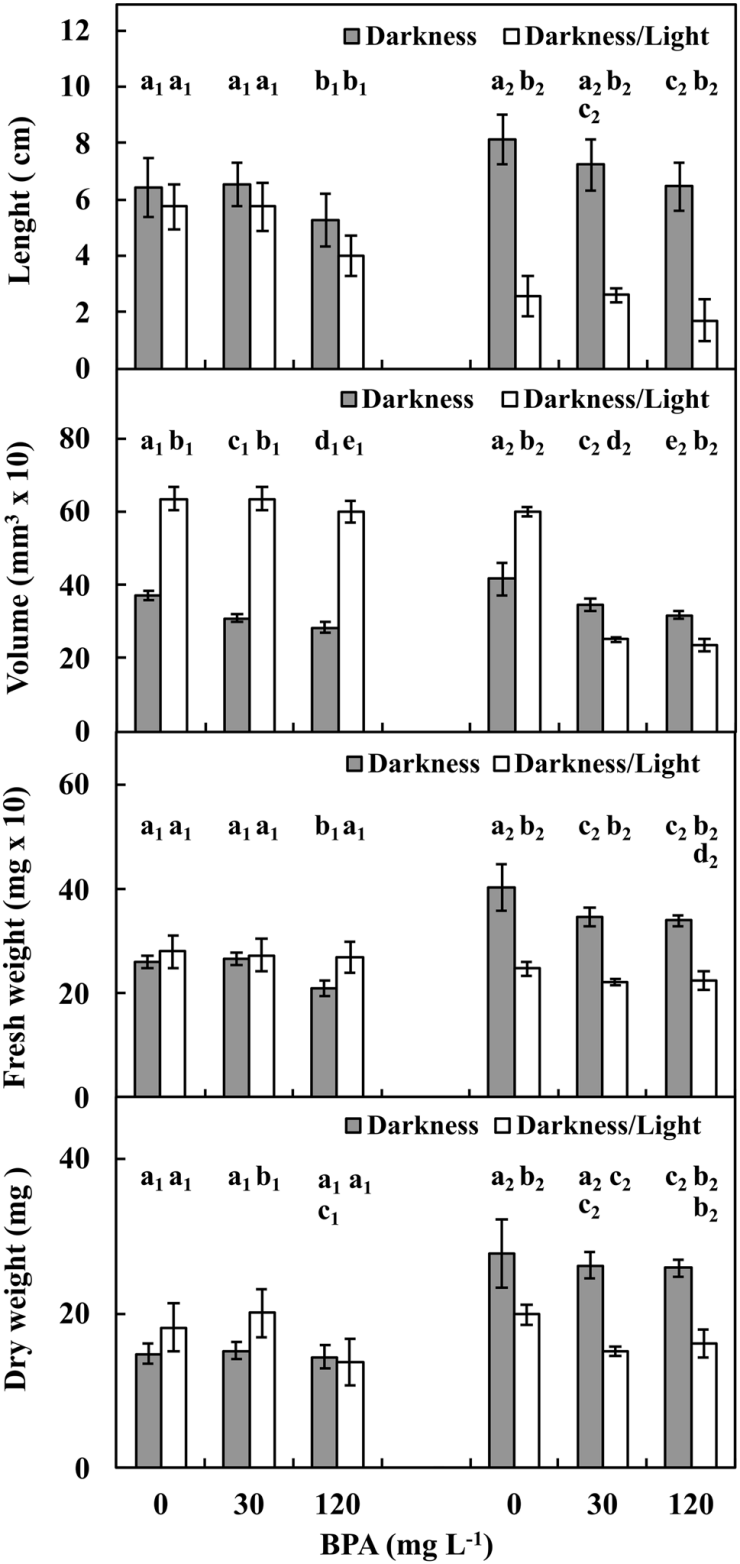
Length (**A**), volume (**B**) as well as fresh (**C**) and dry (**D**) weight of whole roots and stems of *V*. *faba* ssp. *minor* seedlings cultured for 72 h in the darkness and in the light under Ctrl (0 mg BPA) conditions or with 30 or 120 mg BPA L^−1^. Identically labelled columns indicate results that are not significantly different at *p* ≤ 0.05 for darkness and darkness/light groups, separately.

BPA influenced the length, volume, fresh and dry weights of roots and stems (Supplementary Fig. S1A,B; Fig. 4A–D) of *V. faba* ssp. *minor* seedlings cultured under DK and DK/LT conditions.

The lengths of roots in the Ctrl and 30 mg L^−1^ BPA treatments were similar (*p* > 0.05) in both the DK and DK/LT groups and were on average 6.5 cm and 5.5 cm, respectively. However, upon exposure to 120 mg L^−1^ BPA, the roots in the DK and DK/LT treatments were reduced by approximately 30% (*p* ≤ 0.05) compared to those in the other treatments. The lengths of stems in the Ctrl and 30 mg L^−1^ BPA groups were also similar (*p* > 0.05) in both the DK and DK/LT groups and were on average 7.5 cm and 2.5 cm, respectively. However, upon exposure to 120 mg L^−1^ BPA, the stems in both the DK and DK/LT groups were reduced by approximately 25% (*p* ≤ 0.05) compared to those in the other groups. Regardless of BPA variants, the average length of the stems in the DK was threefold greater (*p* ≤ 0.05) than that in the DK/LT (Fig. 4A).

The volumes of roots in the DK groups exposed to 30 and 120 L^−1^ mg BPA were on average 300 mm^3^ and 20% reduced (*p* ≤ 0.05) compared with those in the Ctrl. However, in the DK/LT groups, the volumes of roots were similar to those in the Ctrl (*p* > 0.05) and were on average 600 mm^3^ and twofold increased (*p* ≤ 0.05) than those cultivated in the DK. The volumes of stems in the DK groups treated with 30 and 120 mg L^−1^ BPA were similar (*p* > 0.05) and were on average 320 mm^3^ and 20% reduced (*p* ≤ 0.05) compared to Ctrl. In contrast, the volumes of stems in the DK/LT exposed to the same conditions were on average 240 mm^3^ and approximately 60% reduced (*p* ≤ 0.05) compared to Ctrl (Fig. 4B).

The FW of roots in DK in 120 mg L^−1^ BPA was approximately 210 mg and by approximately 20% (*p* ≤ 0.05) reduced, compared to Ctrl plants and those exposed to 30 mg L^−1^ BPA. In the DK/LT group, the FWs in all variants were similar (*p* > 0.05) and were 270 mg on average. The FWs of stems in the DK and DK/LT groups exposed to 30 and 120 mg L^−1^ BPA were similar (*p* > 0.05) and were 340 mg and 220 mg on average, respectively. These values were also reduced (*p* ≤ 0.05) by approximately 15% and 10%, respectively, compared to Ctrl (Fig. 4C).

DWs of roots in the DK in all series were similar (*p* > 0.05) at 15 mg on average. In the DK/LT groups exposed to Ctrl and 30 mg L^−1^ BPA, similar (*p* > 0.05) values were also obtained, on average 18 mg. However, these values were increased by approximately 20% (*p* ≤ 0.05) compared to the plants exposed to 120 mg L^−1^ BPA. The DWs of stems in the DK in all series were similar (*p* > 0.05), 25 mg on average. In the DK/LT groups, the DWs of the plants exposed to 30 and 120 mg L^−1^ BPA were also similar, 15.5 mg on average, and these values were reduced (*p* ≤ 0.05) by approximately 30% compared to Ctrl (Fig. 4D).

### Phenol and peroxide levels in plant materials and treatment solutions

**Figure 5:**
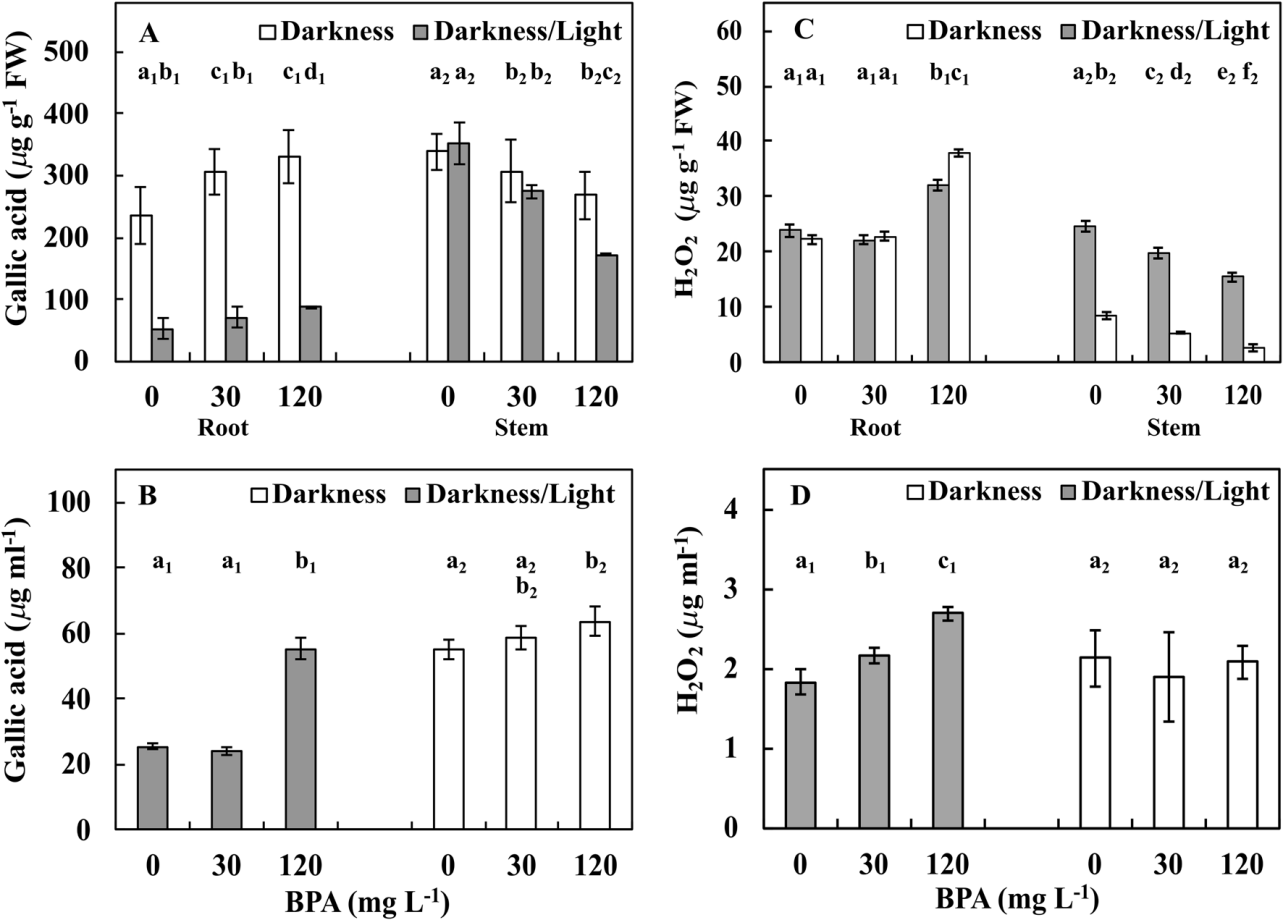
Amounts polyphenols (**A**,**B**) and hydrogen peroxide (**C**,**D**) in the one-third of apical parts of roots and stems (**A**,**C**) and in the treatment solutions (**B**,**D**) of *V. faba* ssp. *minor* seedlings cultured in the darkness and in the darkness/light under Ctrl (0 mg BPA) conditions or with 30 and 120 mg L^−1^ BPA for 72 h. Identically labelled columns indicate results that are not significantly different at *p* ≤ 0.05 for darkness and darkness/light groups, separately.

The amount of total phenols, which is expressed as the amount of gallic acid as a marker, increased (*p* ≤ 0.05) from approximately 235 and 50 µg mL^−1^ in the Ctrl group to approximately 330 and 90 µg mL^−1^ with 120 mg L^−1^ BPA treatment in one-third of roots in the DK and DK/LT groups, respectively. Under the same conditions, the level of total phenols in stems decreased (*p* ≤ 0.05) from approximately 340 and 350 µg mL^−1^ in the Ctrl group to approximately 270 and 170 µg mL^−1^ with 120 mg L^−1^ BPA treatment in the DK and DK/LT groups, respectively (Fig. 5A). In treatment solution from the DK group, the amount of phenols increased (*p* ≤ 0.05) from approximately 25 µg mL^−1^ with Ctrl and 30 mg L^−1^ BPA treatments to approximately 55 µg mL^−1^ with 120 mg L^−1^ BPA treatment. However, in the DK/LT group, the amount of phenols increased (*p* ≤ 0.05) from approximately 55 µg mL^−1^ with Ctrl and 30 mg L^−1^ BPA treatments to approximately 65 µg mL^−1^ with 120 mg L^−1^ BPA treatment (Fig. 5B).

Measurements of peroxides (PXs) calculated based on hydrogen peroxide showed similar amounts per gram of FW in Ctrl roots and those cultured with 30 mg L^−1^ BPA in both lighting conditions (*p* > 0.05; approximately 23 µg g^−1^ FW). However, upon treatment with 120 mg L^−1^ BPA, the levels increased (*p* ≤ 0.05) by 45% and 65% in the DK and DK/LT groups, respectively. In stems of plants in the DK group under Ctrl conditions, approximately 23 µg g^−1^ FW PX was observed. PX levels were reduced (*p* ≤ 0.05) by approximately 20% and 40% respectively, upon treatment with 30 and 120 mg L^−1^ BPA. However, in the DK/LT conditions, the amount of PXs in Ctrl was approximately 8.5 µg g^−1^ FW, and PX levels were reduced (*p* ≤ 0.05) by approximately 20% and 40%, respectively, in the other two series. Moreover, PX levels in the latter series were on average threefold reduced (*p* ≤ 0.05) compared with those in the Ctrl group (Fig. 5C). In treatment solution from the DK group, the amount of PXs increased (*p* ≤ 0.05) from approximately 2 µg mL^−1^ with Ctrl by approximately 15% and 30% upon treatment with 30 and 120 mg L^−1^ BPA treatment. However, in the DK/LT group, the amount of PXs in Ctrl, 30 and 20 mg L^−1^ BPA treatments were similar (*p* > 0.05) and were approximately 2 µg mL^−1^ (Fig. 5D).

### Quinone levels in plant materials and treatment solutions

The amount of 1,4-BQ, one of five types of the studied QSs, in roots decreased both in 30 mg L^−1^ BPA and in 120 mg L^−1^ BPA variants. In the former in the DK and DK/LT it decreased by approximately 30% and 20%, respectively and in the latter by approximately 70% and 40% compared to Ctrl conditions, where the amounts were about 34 and 24 µg g^−1^ FW.

**Figure 6:**
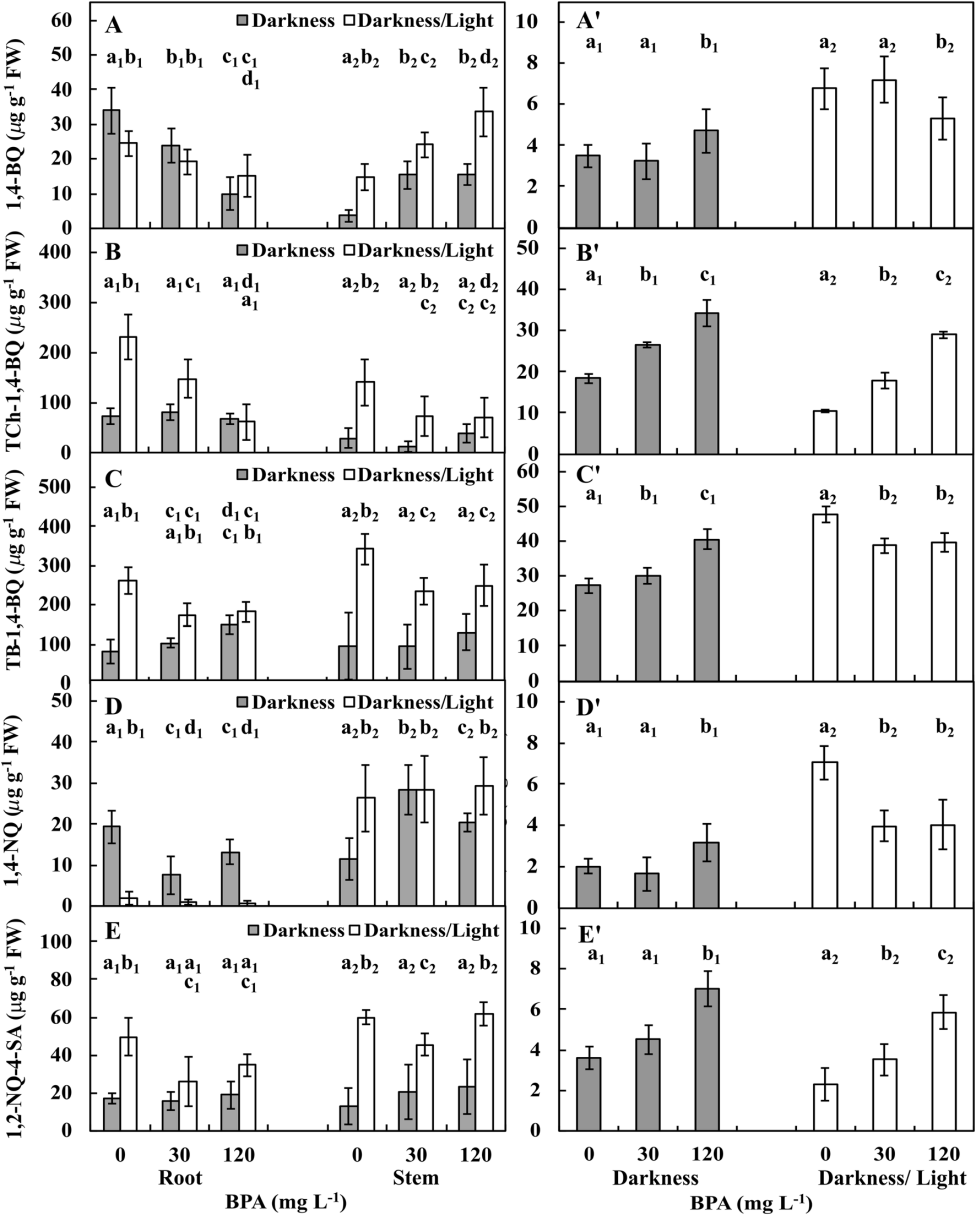
Amounts of quinones 1,4-bezoquinone (**A**,**A′**), tetrachloro-1,4-benzoquinone (**B**,**B′**), tetrabromo-1,4-benzoquinone (**C**,**C′**), 1,4-naphthoquinone (**D**,**D′**) and 1,2-naphtohoquinone-4-sulfonic acid (**E**,**E′**) in one-third of the apical parts of roots and stems (**A**–**E**) and the treatment solutions (**A′**–**E′**) of *V. faba* ssp. *minor* seedlings cultured in the dark and light under Ctrl (0 mg BPA) conditions or with 30 and 120 mg L^−1^ BPA for 72 h. Identically labelled columns indicate results that are not significantly different at *p* ≤ 0.05 for roots and stems (**A**–**E**) as well as for the darkness and darkness/light groups (**A**–**E′**), separately.

The amount of 1,4-BQ in stems in the DK and DK/LT groups increased (*p* ≤ 0.05) from approximately 4 and 15 µg g^−1^ FW, respectively, with Ctrl treatment to approximately 80% and 40%, respectively, with 30 mg L^−1^ BPA treatment. The amount of 1,4-BQ in stems treated with 120 mg L^−1^ BPA did not increase in the DK group and increased in DK/LT by approximately 65% in the DK/LT group. In the treatment solutions, in the Ctrl and 30 mg L^−1^ BPA treatment in the DK group, approximately 3.5 µg g^−1^ FW of 1,4-BQ was noted. While in 120 mg L^−1^ BPA treatment its level increased (*p* ≤ 0.05) by approximately 25% (Fig. 6A,A′).

The amount of TCh-1,4-BQ in roots in DK was not affected by BPA and was on average 75 µg g^−1^ FW. In the DK/LT group, the amount of TCh-1,4-BQ decreased (*p* ≤ 0.05) by 60% and 180% upon exposure to 30 and 120 mg L^−1^ BPA compared with the approximate value of 230 µg g^−1^ FW observed under Ctrl conditions. In stems in the DK group, the amount of TCh-1,4-BQ was reduced (*p* ≤ 0.05) by 200% upon exposure to 30 mg L^−1^ BPA, but the result was not statistically significant (*p* > 0.05). In addition, exposure to 120 mg L^−1^ BPA resulted in a 75% reduction in TCh-1,4-BQ levels. In the DK/LT group, the amount of TCh-1,4-BQ decreased (*p* ≤ 0.05) by approximately 100% upon exposure to 120 mg L^−1^ BPA from the baseline value of approximately 140 µg g^−1^ FW under Ctrl conditions (Fig. 6B,B′).

The amount of TB-1,4-BQ in roots in the DK group increased (*p* ≤ 0.05) by approximately 20% and approximately 50% in plants exposed to 30 mg L^−1^ and 120 mg L^−1^ BPA, respectively, compared to Ctrl. In the DK/LT group, the amount of TB-1,4-BQ fluctuated depending on the BPA concentration and decreased from approximately 260 µg g^−1^ FW upon Ctrl treatment by approximately 50% and 55% in plants exposed to 30 and 120 mg L^−1^ BPA, respectively. In the stems in the DK group, the amount of TB-1,4-BQ was similar among all treatments (average 106 µg g^−1^ FW). However, in the DK/LT group, the amount of this quinone was reduced (*p* ≤ 0.05) by approximately 40% upon treatment with 30 and 120 mg L^−1^ BPA. In the treatment solution, the amount of TB-1,4-BQ in the DK group treated with 30 mg L^−1^ and 120 mg L^−1^ BPA increased (*p* ≤ 0.05) by approximately 45% and 85%, respectively, compared to Ctrl (Fig. 6C,C′).

The amount of 1,4-NQ in roots in the DK group exposed to Ctrl conditions was approximately 19.5 mg^−1^ FW. Upon exposure to 30 and 120 mg L^−1^ BPA, the levels were reduced (*p* ≤ 0.05) by approximately 155% and 46% compared to Ctrl, respectively. In the DK/LT group, approximately 2 mg^−1^ FW of 1,4-NQ was observed. Upon treatment with 30 BPA mg L^−1^, similar (*p* > 0.05) levels were observed, whereas the levels were reduced (*p* ≤ 0.05) on average by 150% upon treatment with 120 mg L^−1^ BPA compared to Ctrl. In the stems in the DK group, the amount of 1,4-NQ was increased (*p* ≤ 0.05) by approximately 150% and by 80% in plants exposed to 30 and 120 mg L^−1^ BPA, respectively, compared to Ctrl (Fig. 6D).

In the treatment solutions, 1,4-NQ levels (approximately 2 mg^−1^ FW) were similar (*p* > 0.05) in the DK groups exposed to 30 mg L^−1^ BPA or Ctrl conditions. However, upon treatment with120 mg L^−1^ BPA, these levels increased (*p* ≤ 0.05) by approximately 50%. In the DK/LT group, similar levels of this QS were noted upon exposure to 30 and 120 mg L^−1^ BPA (*p* > 0.05). On average, these values were reduced by approximately 40% compared to Ctrl (7 mg^−1^ FW; Fig. 6D′).

The levels of 1,2-NQ-4-SA in roots in the DK group were similar with all treatments (average 17 mg^−1^ FW). However, in the DK/LT group, approximately 50 mg^−1^ FW of this QS was recorded under Ctrl conditions. Upon exposure to 30 and 120 mg g L^−1^ BPA, similar levels of 1,2-NQ-4-SA (*p* > 0.05) (average 30 mg^−1^ FW) were observed. However, these levels were reduced (*p* ≤ 0.05) by approximately 60% compared to Ctrl. In stems in the DK group, the amounts of 1,2-NQ-4-SA were similar (average 19 µg g^−1^ FW). However, in the DK/LT group exposed to Ctrl and 120 mg L^−1^ BPA treatment, similar values (60 µg g^−1^ FW) were obtained. Moreover, these values were increased (*p* ≤ 0.05) by approximately 35% compared to treatment with 30 mg BPA mg L^−1^ BPA (Fig. 6E).

In the treatment solution, similar 1,2-NQ-4-SA levels were noted in the DK group exposed to Ctrl and 30 mg L^−1^ BPA (average 4 µg g^−1^ FW). However, upon exposure to 120 mg L^−1^ BPA, these levels increased (*p* ≤ 0.05) by approximately 70%. In the DK/LT group exposed to Ctrl and 30 mg L^−1^ BPA, similar, 2-NQ-4-SA levels were noted (average 3 µg g^−1^ FW). However, upon exposure to 120 mg L^−1^ BPA, the levels increased (*p* ≤ 0.05) by approximately 100% (Fig. 6E′).

### Sugar and protein levels in plant material

**Figure 7:**
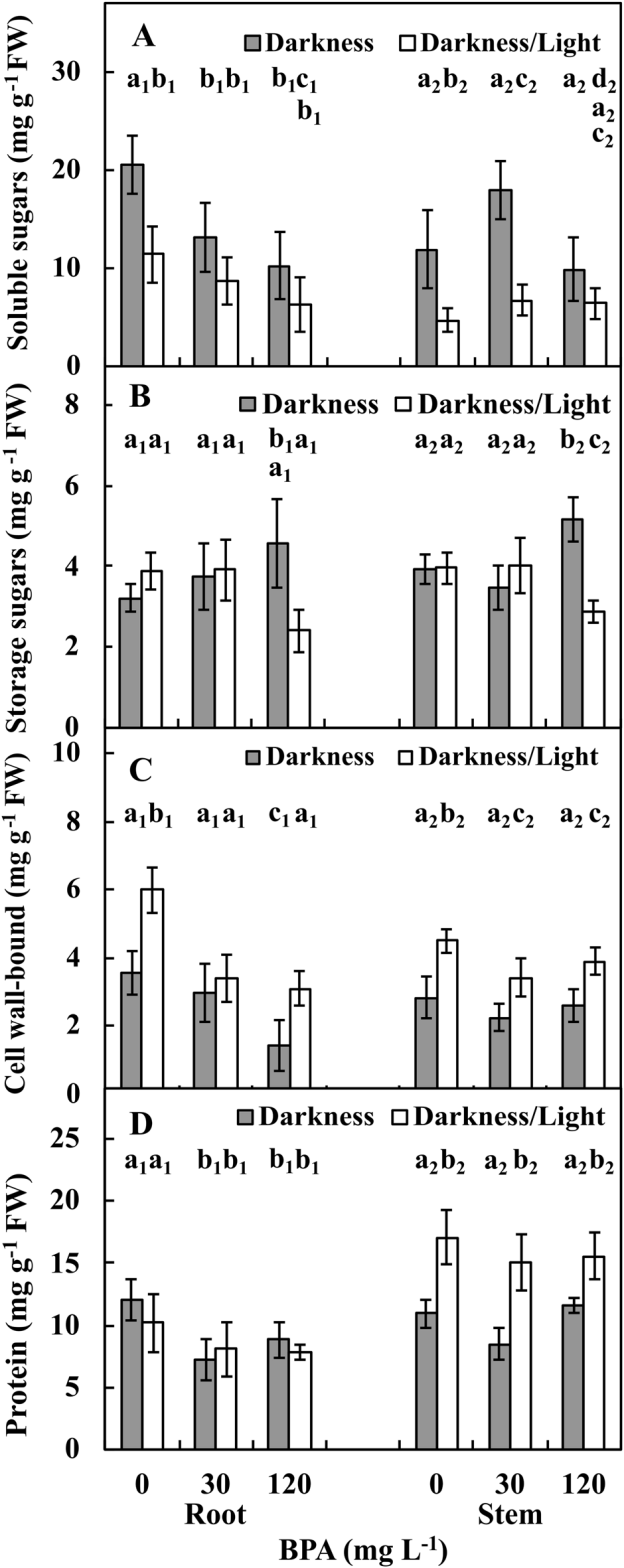
Amounts of soluble (**A**), storage (**B**), cell wall-bound (**C**) sugars and proteins (**D**) in one-third of the apical parts of roots and stems of *V. faba* ssp. *minor* seedlings cultured for 72 h in darkness or darkness/light under Ctrl (0 mg BPA) conditions or with 30 and 120 mg L^−1^ BPA. Identically labelled columns indicate results that are not significantly different at *p* ≤ 0.05, separately.

The levels of soluble sugars (SOS; Fig. 7A) in one-third of each apical part of roots in the Ctrl in the DK and DK/LT were approximately 20 mg and 11 mg g^−1^ FW, respectively, and the difference was significant (*p* ≤ 0.05). Upon treatment with 30 and 120 mg L^−1^ BPA, SOS levels were reduced (*p* ≤ 0.05) by approximately 40% and approximately 60%, respectively, in the DK group and by approximately 25% and approximately 50% (*p* ≤ 0.05), respectively, in the DK/LT group compared to Ctrl. However, average SOS levels in roots in the DK group were 14.5 mg g^−1^ FW after exposure to BPA, and these levels were approximately twofold greater (*p* ≤ 0.05) than those in the DK/LT. SOS levels in one-third of each of the apical parts of stems in the DK and DK/LT groups exposed to Ctrl conditions were approximately 12 mg and 5 mg g^−1^ FW, respectively. The difference was significant (*p* ≤ 0.05). In the DK group, upon treatment with 30 and 120 mg L^−1^ BPA, SOS levels were increased by approximately 40% (*p* ≤ 0.05) and reduced (*p* ≤ 0.05) by approximately 10%, respectively. However, in the DK/LT group, similar SOS levels in response to treatment with 30 and 120 mg L^−1^ BPA were noted compared with Ctrl conditions. However, on average, 13.0 mg g^−1^ FW SOS was noted in the stems of the DK group, and this value was twofold greater (*p* ≤ 0.05) that that noted in the DK/LT group.

The levels of storage sugars (STS; Fig. 7B) in one-third of each apical part of roots in the Ctrl and 30 mg L^−1^ BPA treatments in both the DK and DK/LT groups were similar at 3.5 mg g^−1^ FW (*p* > 0.05) on average. However, upon treatment with 120 mg L^−1^ BPA in the DK group, this parameter was increased by approximately 25%. However, in the DK/LT group, this parameter was reduced (*p* ≤ 0.05) by 50% compared to Ctrl and 30 mg L^−1^ BPA treatments. The levels of STSs in one-third of each apical part of stems subject to Ctrl and 30 mg L^−1^ BPA treatment both in the DK and DK/LT groups were similar at 4 mg g^−1^ FW on average. However, upon treatment with 120 mg L^−1^ BPA in the DK group, this parameter was increased (*p* ≤ 0.05) by approximately 20%. However, in the DK/LT group, this parameter was reduced (*p* ≤ 0.05) by approximately 20% compared to Ctrl and 30 mg L^−1^ BPA treatments in both the DK and DK/LT groups.

The amounts of cell wall-bound sugar (CWS; Fig. 7C) in one-third of each apical part of roots in the DK and DK/LT groups subject to Ctrl conditions were approximately 3.5 mg and 6 mg g^−1^ FW, respectively. The difference was significant (*p* ≤ 0.05). Similar CWS levels were noted between 30 mg L^−1^ BPA and Ctrl in the DK group were noted (*p* > 0.05). However, in the DK/LT group, CWS levels were reduced (*p* ≤ 0.05) by approximately 50% compared to those in the Ctrl in the DK and DK/LT groups, respectively. CWS levels (Fig. 7C) in one-third of each apical part of stems in Ctrl in the DK and DK/LT groups were approximately 3.0 and 4.5 mg g^−1^ FW, respectively. The difference was significant (*p* ≤ 0.05). CWS levels in the DK and DK/LT groups exposed to 30 mg L^−1^ BPA treatment were similar to those noted induced with 120 L^−1^ mg BPA and Ctrl treatments (*p* > 0.05).

The amounts of total proteins in one-third of each apical part of roots in Ctrl in the DK and DK/LT were approximately 13 mg and 11 mg g^−1^ FW, respectively, and the difference was not significant (*p* > 0.05). The protein levels in both the 30 and 120 mg L^−1^ BPA groups were reduced (*p* ≤ 0.05) by approximately 30% compared to Ctrl in both the DK and DK/LT groups. On average, protein levels in the DK and DK/LT groups were similar (*p* > 0.05). The total amounts of proteins in one-third of each apical part of stems in the DK and DK/LT groups with Ctrl treatment were approximately 10 and 16 mg g^−1^ FW, respectively, and the difference was significant (p ≤ 0.05). Protein levels in the DK and DK/LT groups exposed to 30 and 120 mg L^−1^ BPA treatment were similar (*p* > 0.05) to Ctrl. Protein levels in stems in both BPA treatments in the DK group were reduced (*p* ≤ 0.05) by approximately 30% compared to Ctrl.

### BPA levels in culture solutions and plant materials

**Figure 8:**
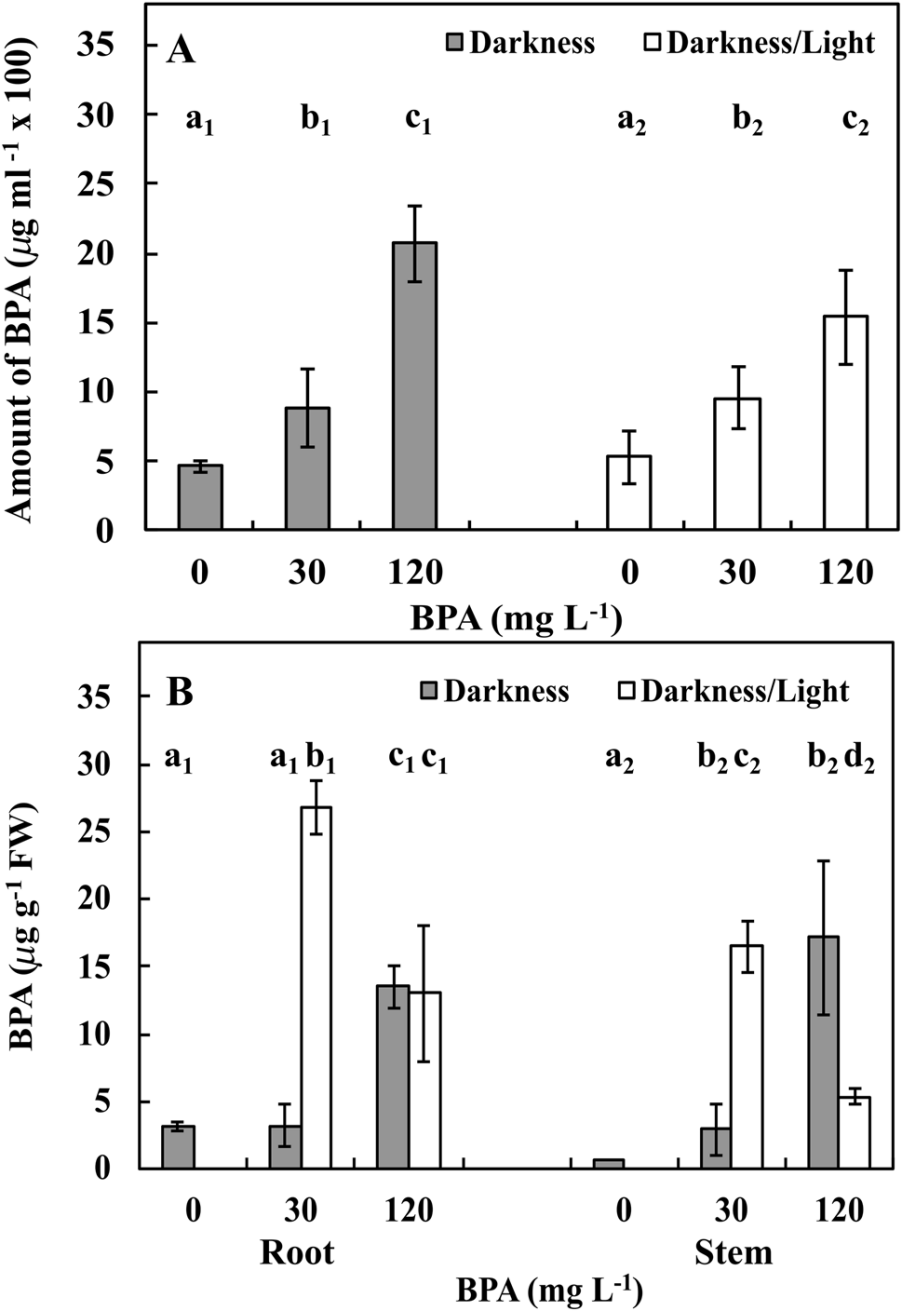
Amount of BPA in the treatment solutions (**A**) as well as in one-third of the apical parts of in roots and stems (**B**) of the *V*. *faba* ssp. *minor* seedlings cultured for 72 h in the darkness and in the darkness/light under Ctrl (0 mg) conditions or with 30 and 120 mg L^−1^ BPA. Identically labelled columns indicate results that are not significantly different at *p* ≤ 0.05 for darkness and darkness/light groups, separately.

In the DK and DK/LT groups exposed to Ctrl conditions, approximately 450 µg and 530 µg BPA was present in the remaining culture solutions, respectively (Fig. 8A). BPA levels were increased (*p* ≤ 0.05) by approximately 50% upon exposure to 30 mg L^−1^ BPA in both the DK and DK/LT groups. However, upon exposure to 120 mg L^−1^ BPA, BPA levels were increased by approximately 120% and approximately 60% (*p* ≤ 0.05) in the DK and DK/LT groups, respectively, compared to Ctrl. When BPA levels induced by Ctrl conditions in the DK group were subtracted from the data obtained upon exposure to 30 and 120 mg L^−1^ BPA, the BPA levels were approximately 420 µg and 1600 µg, respectively. In the DK/LT group, the levels were approximately 420 µg and 1000 µg, respectively. Approximately 750 µg and 3000 µg of BPA were added to the culture solutions upon treatment with 30 and 120 mg L^−1^, respectively. After subtracting the amounts of BPA determined in the culture solutions upon treatment, in the DK groups with 30 and 120 mg L^−1^ BPA treatment, approximately 50% of BPA disappeared. However, in the DK/LT groups treated with 30 and 120 mg L^−1^ BPA, approximately 50% and approximately 70% of BPA, respectively, disappeared.

In the roots and stems of the DK and DK/LT groups subject to Ctrl and 30 mg L^−1^ BPA treatment, the amounts of BPA were similar and were approximately 2.5 µg g^−1^ FW on average. Upon treatment with 120 mg L^−1^ BPA, BPA levels were increased fivefold compared with (*p* ≤ 0.05) that in the other two BPA treatments (Fig. 8B). In the DK/LT group exposed to Ctrl conditions, BPA was not found in roots or stems. Upon treatment with 30 mg L^−1^ BPA, BPA levels were approximately 25 µg and 15 µg g^−1^ FW, respectively. However, upon 120 mg L^−1^ BPA treatment, these levels were two- and threefold reduced (*p* ≤ 0.05) in roots and stems, respectively, compared to 30 mg L^−1^ BPA (Fig. 8B).

### Correlation analysis

Pearson’s correlations analyses between increasing BPA concentrations versus length, volume, FW and DW of roots and stems as well as versus amounts of sugars and proteins, versus Chlls, versus necrosis and phenols, versus peroxides and gallic acids, versus necrosis and peroxides, versus peroxides and endogenous amounts of BPA, versus amount of quinones, necrosis, versus phenols and quinones of seedlings cultured in the DK and DK/LT were performed.

**Figure 9:**
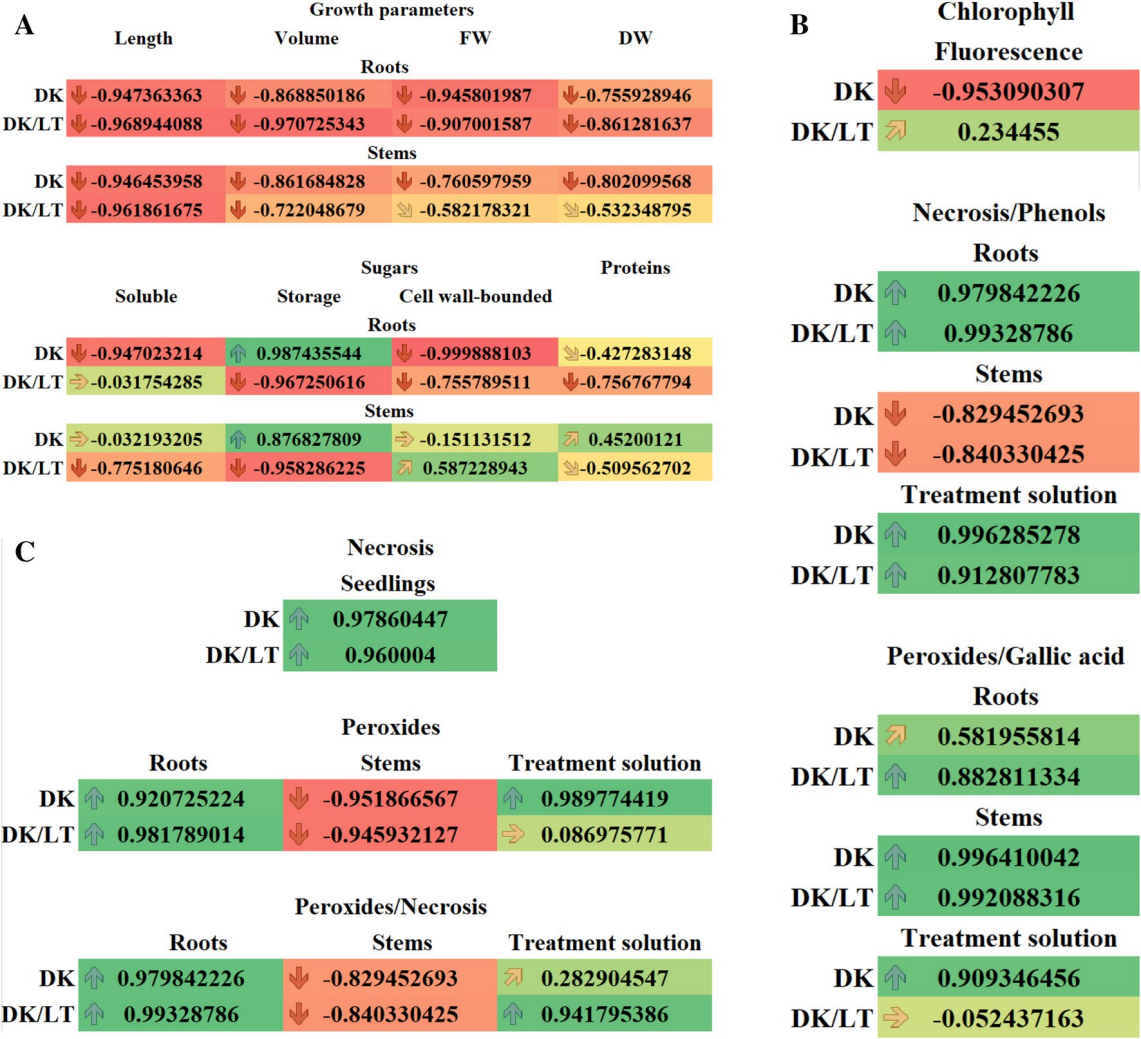

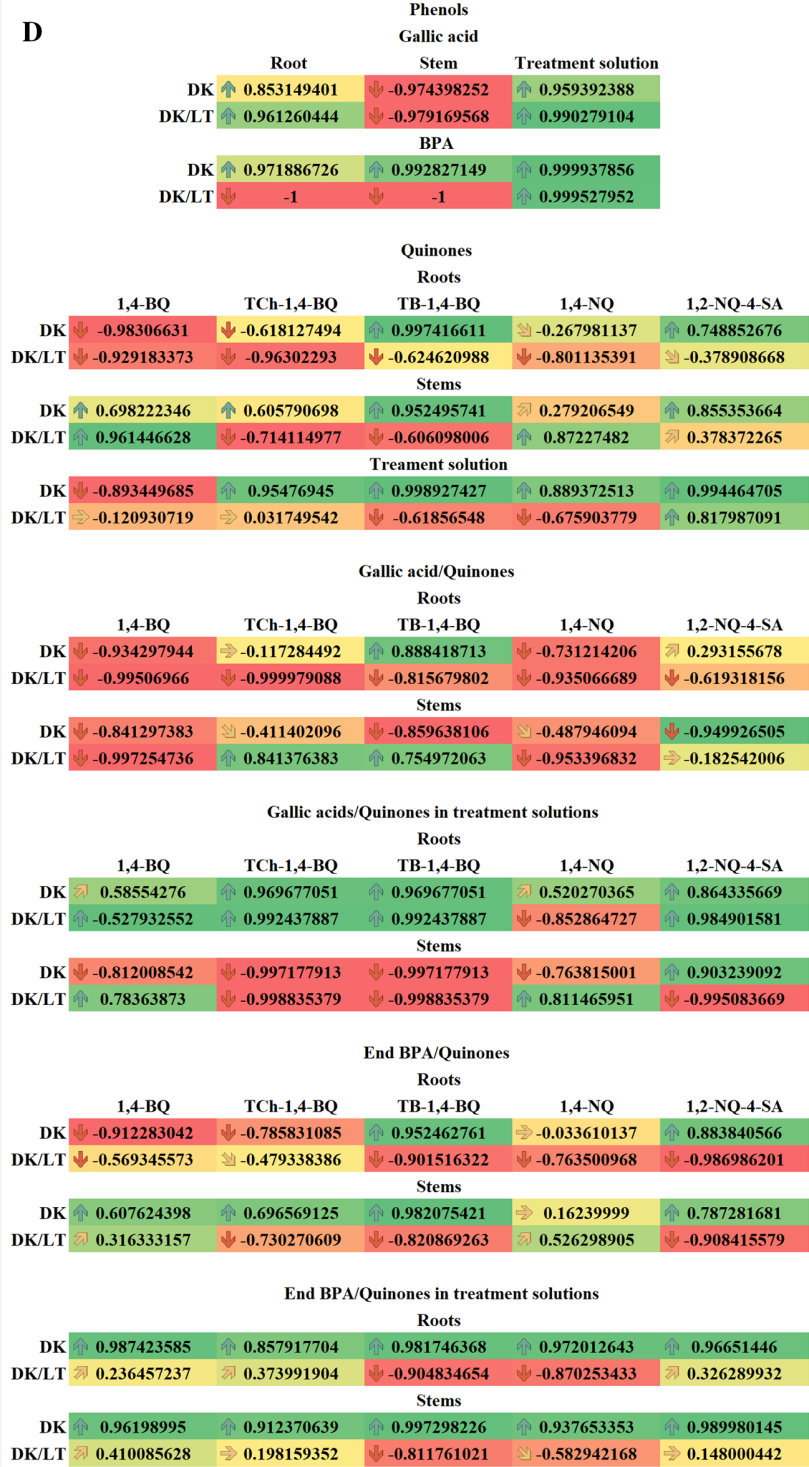
(**A**) Pearson correlation coefficients (*r*_*x,y*_) for increasing concentrations of BPA versus length, volume, fresh weight (FW), dry weight (DW) of roots and stems, and amounts of soluble, storage and cell wall-bounded sugars and proteins in roots and stems in the dark (DK) and the dark/light (DK/LT) groups. (**B**) Pearson correlation coefficients (*r*_*x,y*_) for increasing concentrations of BPA *verus* fluorescence of Chlls in leaf discs; for number of necrotic seedlings versus phenols from roots, stems and treatment solutions; for peroxides from roots, stems and treatment solutions versus gallic acid from roots, stems and treatment solutions;. In the darkness (DK) and in the darkness/light (DK/LT) groups. (**C**) Pearson correlation coefficients (*r*_*x,y*_) for increasing concentrations of BPA versus number of necrotic seedlings (necrosis), the amount of peroxides in roots, stems and treatment solutions as well as for amounts of peroxides in roots, stems and treatment solutions versus numbers of necrotic seedlings. In the darkness (DK) and in the darkness/light (DK/LT) groups. (**D**) Pearson correlation coefficients (*r*_*x,y*_) for increasing concentrations of BPA versus the amount of phenols (gallic acid); for applied increasing concentrations of BPA versus endogenous BPA; for applied increasing concentrations of BPA versus quinones in roots, stems and treatment solutions; for amounts of gallic acid in roots, stems and treatment solutions versus quinones in roots, stems and treatment solutions; for endogenous (End) BPA in roots and stems and treatment solutions versus quinones in roots and stems and treatment solutions. In the darkness (DK) and in the darkness/light (DK/LT) groups.

To facilitate identification of correlative patterns, we conducted a systematic analysis of Pearson’s correlation coefficients between some of the quantitative readouts. The results were visualised using the conditional formatting of MS Excel (Fig. 9A–D).

Increasing concentrations of BPA (0, 30 and 120 mg L^−1^) versus length, volumes, FW and DW of roots and stems of the seedlings cultured in the DK and DK/LT were negatively statistically significant (values lower than 0.4). Similar correlations were observed in roots of the seedlings cultured in the DK, versus soluble, cell wall-bounded sugars and proteins and in the DK/LT, versus the amounts of storage, cell wall-bounded sugars and proteins. In roots in the DK and stems in the DK/LT versus storage sugars and in the DK versus proteins the correlations were positively statistically significant (values greater than 0.4). In the other cases of these groups the correlations were statistically not significant (Fig. 9A).

In leaf discs, the Pearson’s correlation coefficients (*r*_*x,y*_) for increasing concentrations of BPA versus Chlls fluorescence in the DK were negatively statistically significant while in the DK/LT were not significant. The relationships of number of necrotic seedlings cultured in DK and DK/LT in increasing concentration of BPA versus amount of phenols in roots and treatment solution or in stems were respectively positively and negatively significant. The similar correlations were observed in roots and stems in the DK and DK/LT and in the treatment solutions in the DK peroxides versus gallic acid. In the DK/LT the correlations between peroxides and gallic were not significant (Fig. 9B).

In roots, Pearson’s correlation coefficients (*r*_*x,y*_) for increasing concentrations of BPA versus number of necrotic seedlings and amount of peroxides in the DK and DK/LT and amount of peroxides in treatment solution in the DK were statistically significant, while in the DK/LT were statistically not significant, however in stems in the DK and DK/LT the relationships versus peroxides were negatively statistically significant. In roots in the DK and DK/LT and treatment solution in the DK/LT, the relationships of the amounts of peroxides versus number of necrotic seedlings were positively significant. For stems the same relationships were negatively significant (Fig. 9C).

In roots and treatment solution or in stems in the DK and DK/LT, the Pearson’s correlation coefficients (*r*_*x,y*_) for increasing concentrations of BPA versus gallic acid were respectively positively and negatively significant. In roots and stems in the DK and in treatment solution in the DK and DK/LT versus endogenous BPA the relationships were positively significant, while versus roots and stems were negatively significant (Fig. 9D).

In roots, Pearson’s correlation coefficients of seedlings growing in increasing concentrations of BPA versus amount of quinones such as 1,4-BQ and TCh-1,4-BQ in the DK as well as 1,4-BQ, TCh-1,4-BQ, TB-1,4-BQ and 1,4-NQ in the DK/LT were negatively significant. In other cases of these groups the relationships were not significant. In stems in the DK versus 1,4-BQ, TCh-1,4-BQ, TB-1,4-BQ and 1,2-NQ-4-SA and in stems in the DK/LT versus 1,4-BQ and 1,4-NQ the relations were positively significant while versus TCh-1,4-BQ and TB-1,4-BQ the relations were negatively significant. In the other cases in these groups the relationships were statistically not significant (Fig. 9D). In treatment solutions in the DK, the relationships of increasing concentrations of BPA versus amounts of quinones such as 1,4-BQ and in the DK/LT versus TB-1,4-BQ and 1,4-NQ were negatively significant while in DK versus amounts of TCh-1,4-BQ, TB-1,4-BQ and 2-NQ-4-SA and in the DK/LT versus amounts of 1,2-NQ-4-SA the relationships were positively significant. In the other cases in these groups the relationships were statistically not significant (Fig. 9D).

In roots of seedlings growing in increasing concentrations of BPA in the DK the relationships between amounts of gallic acid versus the amounts of quinones such as 1,4-BQ and 1,4-NQ, and in the DK/LT versus all studied quinones were negatively significant while in DK versus TB-1,4-BQ the relationships were positively significant. In the other cases in these groups the relationships were statistically not significant. In stems of seedlings growing in increasing concentrations of BPA in the DK the relationships between the amount of gallic acid versus amount of all studied quinones and in the DK/LT versus amounts of 1,4-BQ and 1,4-NQ were negatively significant while in DK/LT versus TB-1,4-BQ and TB-1,4-BQ were positively significant. In the other cases in these groups the relationships were statistically not significant (Fig. 9D).

In roots of seedlings growing in increasing concentrations of BPA in the DK the relationships between amount of gallic acid versus the amount of all studied quinones in treatment solution and in the DK/LT versus TCh-1,4-BQ, TB-1,4-BQ and 1,2-NQ-4-SA were positively significant while versus 1,4-BQ and 1,4-NQ were negatively significant. In stems in the DK the relationships between amount of gallic acid versus the amount of 1,4-BQ, TCh-1,4-BQ, TB-1,4-BQ and 1,4-NQ and in the DK/LT versus TCh-1,4-BQ, TB-1,4-BQ and 1,2-NQ-4-SA were negatively significant while in the DK versus 1,2-NQ-4-SA and in the DK/LT versus 1,4-BQ and 1,4-NQ relationships were positively significant (Fig. 9D).

In roots of seedlings growing in increasing concentrations of BPA in the DK the relationships between the amount of endogenous BPA versus the amounts of 1,4-BQ and TCh-1,4-BQ and in the DK/LT versus all studied quinones were negatively significant. While in the DK the positively significant relationships were noticed between the amount of endogenous BPA versus the amount of 1,2-NQ-4-SA. In the other cases in these groups the relationships were statistically not significant. In stems in the DK, the relationships between the amount of endogenous BPA versus of the amounts of 1,4-BQ, TCh-1,4-BQ, TB-1,4-BQ and 1,2-NQ-4-SA and in the DK/LT versus 1,4-NQ were positively significant. The relationships between the amount of endogenous BPA in the DK/LT versus of the amounts of TB-1,4-BQ, TB-1,4-BQ and 1,2-NQ-4-SA were negatively significant. In the other cases in these groups the relationships were statistically not significant (Fig. 9D).

In roots in the DK, the relationships between the amount of endogenous BPA in treatment solutions of seedlings growing in increasing concentrations of BPA versus all five studied quinones were positively significant. In the DK/LT versus the amounts of TB-1,4-BQ and 1,4-NQ were negatively significant. In the other cases of these groups the relationships were statistically not significant. In stems in the DK the relationships between the amount of endogenous BPA in treatment solutions of seedlings growing in increasing concentrations of BPA versus all five studied quinones were positively significant. While in DK/LT versus of the amount of 1,4-BQ, TB-1,4-BQ and 1,4-NQ were negatively significant. In the other cases in these groups the relationships were statistically not significant (Fig. 9D).

## Discussion

As one of the anthropogenic pollutants of the environment, BPA represents a fast-growing environmental global problem. Large amounts of BPA are released from plastic products, inducing many harmful effects in humans, animals, and plants^12^.

One of the most important aims of our studies was to find the answer to the following question: Would the phytomeliorative and phytosanitative *V. faba* ssp. *minor* plant is enough to be resistant on negative effects, and to be useful to reduce increasing amount of BPA in the environment?

It was indicated by Li et al., that solubility of BPA reached 381 mg L^−1^ at 25 °C-water^20^. Lin et al. in 2017 showed that in China BPA concentrations are highest in the water of Yuyao City river about 0.23–5.7 mg L^−1^, while about 0.12–0.47 mg g^−1^ FW is in aquatic animals and in surface soil^21^, these concentrations were about 0.30–0.40 mg g^−1^, respectively.

These concentrations were lower than those used in the present studies. However, because of the increasing amount of plastic waste, it is not possible to exclude that the amount of the BPA, locally in the environment, will reach the one, indicated in the present paper.

Experiments of the authors of this paper indicated that 120 mg L^−1^ shows maximum freely dissolving BPA during 3 weeks in the dark at 23 °C. Moreover, studies showed that root and stem lengths used as primary testing parameter of *V. faba* ssp. *minor* seedlings at 5, 15 (data not attached) and 30 mg L^−1^ BPA were not affected in statistically important manner compared to the control. While, 60 and 90 mg L^−1^ of BPA (data not attached) reduced the length of roots and stems. However, only 120 mg L^−1^ of BPA induced a statistically important effect compared to Ctrl and 30 mg L^−1^ BPA treatments. We suggested that 120 mg L^−1^ is the final concentration of BPA absorbed by growing plants, thus, BPA concentrations of 30 and 120 mg L^−1^ were used in this study (Fig. 1).

### Effect of bisphenol A on growth, morphology, photosynthetic parameters, and hydrogen peroxide, phenol and quinone levels

Scientific data indicated that 1.5 mg L^−1^ BPA elevated the FW and DW of stems and leaves as well as leaf area in soybean seedlings without affecting the content of Chl *a* and its fluorescence. However, in seedlings treated with 7.0, 12.0, 17.2 and 50.0 mg L^−1^ BPA, a limited net photosynthetic rate as well as altered Chl *a*, Fv/Fm, PSII and ETR values were observed^7^. In *A. thaliana* seedlings, BPA at 1.0 and 5.0 µM elevated FW as well as tap root length and lateral root formation. However, 10.0 and 25.0 µM BPA reduced these parameters in a dose-dependent manner. The effects of BPA on indicating that BPA has no notable role in priming the light response in the germination and early growth of seedlings^6^. *L. culinaris* plant growth and development are strictly related to the amounts and proper activity of chlorophylls, which were significantly decreased after treatment with 50 and 100 mg L^−1^ BPA. However, the amount of Chl *b* in relation to Chl *a* at 10 mg L^−1^ decreased root growth^8,9^.

The results of the present paper confirmed some of the literature results. The growth of *V. faba* ssp. *minor* seedlings was strongly (− 0.81 ≥ *r* ≥ − 1) and moderately (− 0.41 ≥ *r* ≥ − 0.8) inhibited (Fig. 9A) by limiting the length and volume of roots and stems both in DK and DK/LT groups, the FWs of roots and stems in the DK group, and the FWs of roots and stems in the DK/LT group (Fig. 4).

In leaves of cucumber (*C. sativus*) cultivated under LT, BPA elevated amounts of H_2_O_2_, a direct factor leading to inhibition of D1 protein turnover. In addition, BPA induced aggregation of PSII but not PSI and inhibition of CO_2_ assimilation^22^. Rąpała et al.^23^ showed that BPA generally had no effect on the Chl *a*/*b* ratio at concentrations greater than 5 mg^−l^ L^−1^; below this concentration, BPA significantly increased Chl amounts. Similar results were described by Zhang et al.^10^, who studied lettuce, tomato, soybean, maize and rice. These researchers showed that 1.5 and 3.0 mg L^−1^ BPA improved PSII efficiency, increased the absorption and conversion efficiency of primary LT energy and accelerated electron transport in each plant. Moreover, all of these parameters increased photosynthesis^9,10^.

In leaf discs of *V. faba* ssp. *minor* cultivated in DK, the effect of BPA on the amount of Chl was strongly negatively correlated. However, in the DK/LT groups, no significant differences were noted between the groups (Fig. 9B). In the seedlings cultured in DK/LT, only R_fd_ values indicating necrosis formation were negatively correlated with BPA (*r*_*xy*_ = – 0.99).

BPA in *V. faba* ssp. *minor* seedlings (*r*_*xy*_ ≥ 0.96) induced necrosis, leading to the formation of an increased number of injured seedlings and injured area in leaves both in the DK and DK/LT groups (Fig. 9C). This finding was strongly and positively correlated in roots and treatment solutions and negatively correlated in stems with increasing and decreasing amounts of H_2_O_2_ in both lighting conditions as well as both plant tissue and treatment solutions, respectively (Fig. 9D). It was also observed that in the roots of seedlings cultivated under DK and DK/LT conditions, the amount of H_2_O_2_ was moderately and strongly positively correlated with the amount of phenols, respectively (Fig. 9B). However, in stems in both cases, the reduced amount of H_2_O_2_ was strongly and positively correlated with the reduction in the amounts of phenols (Fig. 9B). Increasing BPA concentrations were strongly positively correlated with the number of necrotic seedlings (Fig. 9B). The increasing amount of phenols in the treatment solutions was correlated with the increasing number of necrotic seedlings. This finding indicated that phenols were moved from stems because BPA levels decreased in seedlings; thus, the numbers of necrotic seedlings were strongly positively correlated with the amount of phenols in the treatment solutions under both lighting conditions (Fig. 9B). All of these results indicating interactions between necrosis, phenols and H_2_O_2_ suggested synthesis of QSs from phenols and H_2_O_2_^24^, which was visible as dark brown necrotic dots on the surface and inside roots and stems of faba bean. Analysing these results, one should remember that BPA is also a phenol^1,2^. Increasing concentrations of applied BPA were strongly positively correlated with the amount of BPA in roots, stems and treatment solutions in the DK group. However, in the DK/LT group, a strong positive correlation was observed only for the treatment solutions. It seems that light and peroxides also caused a decrease in this phenol, which can be metabolised to QS.

These results are consistent with the results of Zhang et al.^10^, who showed that the amount of BPA in the roots of 7-days-old *A. thaliana* seedlings increased with increasing BPA concentration. Moreover, cultivation of *A. thaliana* seedlings for 7 days decreased the amount of BPA in roots, indicating that BPA was metabolised by plant tissues.

Thus, we suggest that QSs in *V. faba* ssp. *minor* can be synthesized from endogenous and applied (BPA) phenols. Phenols as well as other antioxidant compounds^25^ not studied in these experiments played protective roles against the toxic effects of H_2_O_2_^26^.

H_2_O_2_ is directly visible in stems under both lighting conditions, where the amounts of phenols and peroxides decreased (Fig. 5). This result indicates that stems are responsible for the metabolism of these compounds.

The amounts of accumulated QS in the plant tissues of faba bean seedlings were moderately and strongly positively correlated with increasing concentrations of BPA applied. Specifically, 1,4-BQ levels were increased in stems under both lighting conditions. TCh-1,4-BQ and 1,2-NQ-4-SA levels were increased in roots and stems in the DK group, and TCh-1,4-BQ and 1,4-NQ levels were increased in stems in the DK and DK/LT groups. In the treatment solutions, strong positive correlations with increasing BPA concentrations were noted for three (TCh-1,4-BQ, TB-1,4-BQ, 1,4-NQ) of five quantified QSs in the DK group and for 1,2-NQ-4-SA under both types of lighting conditions (Fig. 9C). This result indicated that the decreasing amounts of QSs in plant tissue were related to their release to the treatment solutions, especially in the case of TCh-1,4-BQ and 1,2-NQ-4-SA in roots and stems (Fig. 6).

Person’s coefficients suggest that TB-1,4-BQ in roots and stems in the DK and DK/LT group, TCh-1,4-BQ and TB-1,4-BQ in the DK/LT group and 1,2-NQ-4-SA in the DK group were potential products of plant phenol oxidisation present in stems. However, this finding indicates that all QSs can be moved to treatment solutions from roots, whereas 1,4-BQ and 1,4-NQ in the DK and DK/LT groups and 1,2-NQ-4-SA in the DK group can only be move from stems (Fig. 9D).

We propose that BPA represents the substrate for 1,4-BQ and 1,4-NQ in roots and stems in the DK group (Fig. 9D).

However, the BPA as well as the endogenous phenols in the tissues of faba bean might be oxidised to quinones^27^ with the use of the endogenous hydrogen peroxides by one of the well-known enzymes in plants, i.e., polyphenol oxidase. Moreover, Yoshida et al. indicate that BPA was metabolised by enzymes from several species of fruits and vegetables as well as by commercially available ones to monoquinone [4-[1-(4-hydroxyphenyl)-1-methylethyl]-1,2-benzoquinone] and bisquinone [4,4′-(1-methylethylidene)bis(1,2-benzoquinone] derivatives^27^.

The presence of increasing BPA levels in the treatment solution of the seedlings in the BPA treatment groups is obviously noted. However, the presence of BPA in the Ctrl treatment solutions in both the DK and DK/LT groups and in the roots and stems of seedlings cultivated in the DK group indicate that BPA came from the environment, i.e., the laboratory equipment, plastic tubes, deionised water (because the deioniser is made of plastic) and likely the coats of seeds used in the experiments because they were stored in plastic boxes or sacs.

### Effect of BPA on cell death and protein amounts

We concluded that QSs were responsible not only for the dark colour in the necrotic areas (Figs. 1, 6 and 7) but also for toxic effects in roots and stems of faba bean seedlings. The toxicity of QS is related to its interaction with cellular compounds, including proteins^25^. Protein levels were moderately limited by BPA in roots under both lighting conditions and in stems in the DK/LT group (Fig. 9A).

Such effects were observed in other plants, e.g., *A. thaliana*, in which BPA decreased the amount of proteins in leaves^23^. It is known that a decrease in the protein amounts might diminish the amounts and activities of enzymes, receptors and many signalling and metabolic molecules, including proteins that are important for cytochromes and DNA^28^. Proteins also form many conjugates. For example, sugars form lectins, one of the best known glycoproteins of the Fabaceae family^29^, to which *V. faba* ssp. *minor* belongs^14^. In *V. faba* ssp. *minor*, proteins account for 30% of seed FW^14^. One study showed that BPA increased the expression of HSP70-9 (70 kDa heat shock proteins) in spinach^30^ in a concentration-dependent manner.

Thus, in the unaltered areas, living cells were identified. However, in the immediate proximity to the injured regions, the dying cells and dispersed chromatin were observed. In the injured regions, cells had no nuclei. The living cells were clearly separated from the dead cells. These results indicated that BPA did not induce the PCD process, but the cells were probably directly killed by QSs. PCD in plants is a process that occurs via vacuolar or autolytic types of death^31,32^, leads to elimination of damaged parts of cells or whole cells and may induce thickening of cell walls^33,34^ to build a barrier against environmental biotic^35^, abiotic, or endogenous^18^ factors.

### Effect of BPA on sugar levels

Sugars in plants are important molecules. Sugars in the form of glucose serve as sources of energy^36,37^ and substrates for cellulose, hemicelluloses and callose synthesis^38^. Sugars also providing elements of cell wall structure^37,38^ play a signalling role^36,39^.

In roots in the DK group, the concentrations of applied BPA were strongly negatively correlated with the amounts of soluble and cell wall-bound sugars but strongly positively correlated with storage sugars. In the DK/LT group, soluble sugars were not affected, but storage and cell wall-bound sugars were strongly and moderately negatively affected, respectively. This finding indicates that light had a positive effect on the maintenance of soluble sugars in roots. In stems in the DK group, BPA strongly positively affected only storage sugars. BPA had no effect on the amounts of soluble and CWSs. In the DK/LT group, the amounts of soluble sugars were moderately and strongly negatively correlated with the applied BPA (Fig. 9A).

## Conclusions

The literature describes the effects of BPA on the growth and development of plants^10^, but these descriptions are insufficient.

The results of the multilateral studies presented in this paper are divided into a various topics: (i) growth and morphology, (ii) photosynthesis and (iii) metabolism of roots and stems. These topics are assessed in relation to two types of lighting conditions, i.e., DK and DK/LT. Of the two experimental concentrations of BPA employed in this study, i.e., 30 and 120 mg L^−1^, the latter one caused greater negative effects.

The results showed that laboratory equipment and water as well as potentially the coats of seeds contained BPA, which explains why necrotic seedlings also appeared in the Ctrl series. Analyses indicated that BPA accumulated in plants, and the comparative recalculation of these results indicated that roots and stems of *V. faba* ssp. *minor* seedlings exposed to 120 mg L^−1^ BPA and in the DK/LT group metabolised BPA more effectively compared with 30 mg L^−1^ BPA. BPA positively and negatively influenced the amounts of peroxides and phenols, respectively, in roots, stems and treatment solutions. QS levels were variously influenced by BPA, and this effect was dependent on the part of seedlings and lighting conditions. We suggest that QSs in *V. faba* ssp. *minor* can be synthesized from endogenous and applied (BPA) phenols as well. In several cases, these compounds were transported to the culture solutions. The QSs identified in the DK group included TCh-1,4-BQ, TB-1,4-BQ, 1,4-NQ, and 1,2-NQ-4-SA, whereas 1,2-NQ-4-SA was identified in the DK/LT group. It seems that endogenous phenols are responsible for the increased levels of TB-1,4-BQ in roots in the DK group as well as TCh-1,4-BQ and TB-1,4-BQ in stems in the DK/LT group. We proposed that BPA was metabolised to generate all QS in the roots and stems in DK; however, two of these QSs, i.e., TB-1,4-BQ and 1,2-NQ-4-SA are crucial.

Finally, the results of the present research allowed us to conclude that the toxic effect of BPA is directly related to QSs that interact with cellular metabolites, e.g., proteins, and damage stems and roots of faba bean, visible as necrotic changes in both types of lighting conditions in which the dead cells were observed.

## Materials and methods

### Plant material, seed germination, culture, treatment and measurements

Laboratory equipment used to germinate and cultivate seeds and seedlings of *Vicia faba* ssp. *minor* cv. Bobas (Danko, Sobiejuchy 2, 88–400 Żnin, Sobiejuchy 2, Poland; http://www.danko.pl) were autoclaved at 120 °C for 20 min.

The working solution was obtained by mixing the appropriate amount of the BPA in deionised water. The mixture was incubated at 23 ± 1 °C in the darkness to allowing of the chemical to dissolve during 3–4 weeks to imitating natural conditions.

The steps of experiment: (i) germination of 30 seeds in the darkness in Petri dishes (15 cm in diameter and 3 cm high) on two blotting papers with 60 mL of deionized water at 23 ± 1 °C for 3 days; (ii) putting on the bottom of 1-L Erlenmeyer flasks and adding 25 cm^3^ of water (Ctrl, 25 cm^3^) or water solution of 30 and 120 mg L^−1^ BPA; (iii) transfer of fit-looking seedlings with roots 2.5 cm (± 0.3 cm) in length into to the flasks; (iv) covering the necks (5 cm in diameter) with a sheet of aluminium foil (10 × 10 cm); (v) cultivation in DK or DK/LT (250 µmol m^−2^ s^−1^; 12 h/12 h) at 23 ± 1 °C and 92 ± 2% humidity in the flask for 72 h (Fig. 1).

The following analyses were conducted: the number of necrotic seedlings as well as quantitative analyses of volume, fresh and dry weight, flavonoids, hydrogen peroxides, sugars, proteins, QSs and BPA in one-third parts of roots and stems and treatment solutions of *V*. *faba* ssp. *minor* seedlings (Supplementary Fig. S1). Moreover, the Chl fluorescence, PSII activity and necrotic areas of the 1-cm-leaf discs were measured. Nonsterile discs of 2-month-old plants of *V*. *faba* ssp. *minor* cultured in 10-cm sterile Petri dishes with two blotting papers in the DK or DK/LT with 5 mL of water (Ctrl) and 30 or 120 mg L^−1^ BPA for 72 h were used for these analyses.

### Analyses of chlorophyll fluorescence and PSII activity

Chl *a* fluorescence (Fig. 1) in the leaf discs (Supplementary Fig. S2) was assessed under blue LT fluorescence microscopy and using a non-invasive measurement of PSII activity (Fig. 2). For measurements of the total fluorescence of chlorophylls, the leaf discs (Fig. 1) that were cultured as described in the 'Plant material' section were placed on a glass slide with a drop of 50% glycerol in water under the coverslip, and microphotographs were obtained with a 1-s exposure and used to measure the intensity of red fluorescence of Chlls (R.F.I.Ch.).

PSII activities (Fig. 2) were measured using a FluorCAM imaging system (Photon Systems Instruments Ltd., Czech Republic) according to the manual. Three whole seedlings per repetition were dark adapted for 20 min inside the measuring chamber to reach a fully oxidised state of PSII photochemistry. The quantum efficiencies were calculated based on technical parameters. Using this technique, changes in the following photosynthetic parameters were detected in plants cultured in DK/LT: maximal PSII quantum yield (Fv/Fm), efficiency of the PSII adapted state, steady-state PSII quantum yield (QY), steady-state nonphotochemical quenching (NPQ), photochemical quenching (qP) and an empirical parameter used to assess plant vitality (R_fd_).

### Damage analysis based on the number of necrotic seedlings and necrotic areas of leaf discs

To classify faba seedlings cultured in the DK or DK/LT as necrotic (Fig. 3A), the apical, subapical, subbasal and basal parts of roots and stems were observed using a magnifying glass (5×). The changes were documented in images obtained under a stereoscopic microscope (Supplementary Figs. S3 and S4).

Necrotic areas (Fig. 3B) in the leaf discs were quantified based on algorithms of ImageJ software using the photos (Supplementary Fig. S2A–F) obtained under the stereoscopic microscope.

### Quantification of cell viability

To determine cell viability (Supplementary Fig. 5), nonfixed subapical fragments of roots and stems of seedlings were removed, washed twice with 0.01 M sodium phosphate buffer (PHB) at pH 7.4, and stained with a mixture of 100 µg mL^−1^ acridine orange (AO) and ethidium bromide (EB) at (PHB) for 5 min. Then, the fragments were washed twice with (PHB) and fixed with 2% glutardialdehyde (Merck) at PHB for 15 min, cut into very thin longitudinal sections, washed thrice with PHB, and observed and photographed under the blue light of a fluorescence microscope according to the methods described by Kunikowska et al.^18^. The colour of chromatin, which ranged from green to red, correlated with increased fluorescence intensity (RFI). This parameter allowed us to distinguish living, dying and dead cells. Green AO fluorescence in the nuclei indicates a cell with unaltered plasma membrane permeability (i.e., a living cell), whereas red one of EB is only observed in dead cells in which a plasma membrane is completely altered. Green-yellow, yellow, yellow-orange, and orange nuclei indicate cells with partially altered plasma membrane due to the increasing amount of EB which masks green fluorescence of OA. These cells are dying ones and during the studies were not observed. RFI values of less than 35 a.u. and greater than 55 a.u. indicate living and dead cells, respectively, as described in Kunikowska et al.^18^. RFI values between 35 and 55 a.u. indicate cells dying via programmed cell death (PCD)^18^.

### Measurements of length, volumes, and fresh and dry weight of roots and stems

Lengths (Fig. 4A) of whole roots and stems were measured using millimetre paper. Volumes (Fig. 4B) were determined volumetrically using a laboratory glass cylinder. For fresh weight (FW; Fig. 4C) measurements, the roots and stems removed, washed with deionized water, gently dried with filter paper, and weighed to obtain the FW. To measure dry weight (DW; Fig. 4D), the fragments were dried at 80 °C in an oven for 24 h and weighed.

### Determination of phenols, quinones, hydrogen peroxides, sugars and protein levels

One-third of the apical parts of roots or stems of each seedling were washed with deionized water, gently dried using filter paper, weighed, dried at 80 °C for 24 h and homogenised in Tris–HCl buffer (0.005 M, pH 7.45; THB) or in 80% (v/v) methanol (500 µl g^−1^ FW) at 23 °C. The homogenate was centrifuged at 15,000×*g* for 10 min and re-extracted. Supernatants were combined. Homogenates in THB were used for the determination of peroxides (Fig. 5A) and proteins (Fig. 7) with the Coomassie G-250 reagent as described by Bradford^40^. Homogenates in methanol were used to determine phenols (Fig. 5A,B), QSs (Fig. 6) and soluble sugars (SOS; Fig. 7)^41^.

Pellets were evaporated for 30 min at 30 °C to remove methanol, extracted at 4–8 °C with 500 µl g^−1^ FW of 35% (v/v) perchloric acid (HClO_4_; POCH) for 24 h, centrifuged at 15,000×*g* for 20 min and re-extracted. Storage sugars (STS; mostly consisting of starch and hemicelluloses) were determined using the combined homogenates^41^.

The final pellets were supplemented with 500 g^−1^ FW of 67% (v/v) sulfuric acid (H_2_SO_4_; POCH), shaken for 24 h at 4–8 °C and centrifuged at 15,000×*g* for 20 min. Cell wall-bound sugars (CWS; mostly consisting of cellulose and nonstorage hemicelluloses) were determined using the supernatants^37,41^.

The total phenol amounts in the plant extract and treatment solutions were measured according to the method described by Sharma et al.^42^. Specifically, the absorbance of the mixture containing 50 µl of sample, 50 µl of Folin-Ciocalteu reagent (diluted with water, 1:1), 100 µl of Na_2_CO_3_ and 800 µl water was measured at 725 nm. The amount of total phenols was calculated according to the calibration curve prepared using gallic acid as a standard in the range of 1 to 50 µg per sample.

The levels of QSs, i.e., 1,4-bezoquinone (1,4-BQ), tetrachloro-1,4-benzoquinone (TCh-1,4-BQ), tetrabromo-1,4-benzoquinone (TB-1,4-BQ), 1,4-naphthoquinone (1,4-NQ), and 1,2-naphthoquinone-4-sulfonic acid (1,2-NQ-4-SA), were determined using the method described in Zaki et al.^24^ by measuring absorbance against a blank at 675, 460, 380, 690 and 530 nm, respectively, in mixtures containing 100 µl of sample, 300 µl each of rhodamine (0.5%) in methanol (100%) and aqueous ammonia (10%) and 800 µl of methanol (added as the last one after 15 min). The amounts of each QS were calculated according to the molar absorbance coefficient (*ɛ* × 10^–8^), i.e., 11.3, 10.8, 9.8, 14.5 and 6.1.

The peroxides were determined reflectometrically in supernatants or treatment solutions immediately after their preparation using the test strips and RQflex 10 plus system (Merck). The test strips were inserted to the sample in the Eppendorf-like tubes for 2 s, and then after next 15 s, peroxides were indicated on strips by the blue colour after oxygen transfer by peroxidase on organic redox indicator. Their level in mg L^−1^ were measured according to the device-inserted procedure.

The sugars were determined using the anthrone test^41^. The reaction mixture consisted of 0.9 cm^3^ anthrone reagent prepared with 200 mg anthrone in 100 mL 72% v/v sulfuric acid, and 50 µl of the sample was heated at 110 °C in a dry heating block for 11 min and then cooled on ice for 10–15 min. Absorbance was measured at 630 nm. Blank solutions were prepared using respective volumes of methanol, HClO_4_ or H_2_SO_4_ instead of the tested sample. The amount of sugars was estimated using a calibration curve prepared with glucose as a standard in the range of 1 to 50 µg per sample.

### Detection and determination level of bisphenol A levels in the treatment solutions and seedlings

Measurements of BPA (Fig. 8A) levels in the treatment solutions were performed spectrophotometrically. The maximum absorbance of BPA at 279 nm was determined using standards (10, 20, 30, 40, 50 and 60 µg per sample) in 80% methanol by scanning from 250 to 300 nm. Using this protocol, the calibration curve was prepared for the estimation of BPA levels. BPA was isolated from 1 mL of the culture solution with 3, 2 and 1 mL of ethyl acetate. The obtained organic phases were combined, evaporated to dryness at 40 °C using a rotary evaporator, dissolved in 80% methanol, filtered through 0.22-μm Millipore™ filters and used to measure the absorbance.

The isolation method described by Zhang et al.^10^ was used To analyse the amount of BPA in the plant material (Fig. 8B). The whole stems and roots were weighed, freeze dried, ground in Eppendorf tubes with a plastic pestle and extracted by mechanical shaking with pure methanol for 4 h. Then, the mixture was centrifuged at 15,000×*g* for 10 min, and the residues were re-extracted. Supernatants were combined and filtered through 0.22-μm Millipore™ filters and evaporated to dryness at 40 °C using a rotary evaporator. The residue was dissolved in 1 mL of methanol and subjected to chromatography. Separation of 20-µL samples was performed at 30 °C using an isocratic program with the water:acetonitryl (40%:60%; v:v) mixture as a solvent and a flow of approximately 0.8 mL min^−1^ for 10 min. The amounts of BPA in the samples were estimated according to the calibration curve that was generated using BPA as a standard. Control samples containing only water and the indicated amount of BPA were also prepared.

### Statistics and equipment

Three biological replicates, at least in tri-, duplicate and more random samples, were analysed. The samples were prepared from at least six plants. The results of morphologic tests were statistically verified using Student’s *t-test*.

The results of metabolic tests were verified using the Mann–Whitney *U test*, and the *p* value was determined to assess significant differences between the results, using EXCEL software (licenced 365 MS Office) by independent step-by-step analyses of each column of results. Significant differences between the results were observed at *p* ≤ 0.05. Calculations, all charts, and tables were prepared using EXCEL software (licenced 365 MS Office).

The results were also verified using Person’s correlation coefficient. Values of *r*_*xy*_ between 0.0 and 0.4 (from orange to yellow-coloured cells with horizontal and upwards slant arrows) or – (minus) 0.4 (from orange to light salmon-coloured cells with horizontal and downwards slant arrows), 0.41 and 0.8 (from yellow-to-yellow green coloured cells with upwards slant and vertical downwards arrows) or – (minus) 0.8 (from light salmon to dark salmon-coloured cells with downwards slant and vertical downwards arrows) as well as values greater than 0.81 (green coloured cells with vertical arrows) or below – (minus) 0.81 (red coloured cells with vertical downwards arrows) indicate no, moderate, and strong correlations, respectively. Values of *r*_*xy*_ greater than 0.41 were considered statistically significant at *p* ≤ 0.05.

Laboratory equipment was sterilized using Varioclav (https://animalab.pl). The total red fluorescence intensity of Chl (R.F.I.Ch.) in leaf discs and the viability of cells was assessed under an epifluorescence microscope (Optiphot-2, Nikon, Japan) equipped with a B2A filter and ACT-1 software (Precoptic, Poland; https://precoptic.pl). PSII activity was measured with a non-invasive technique using the FluorCAM imaging system (Photon Systems Instruments Ltd., the Czech Republic), whereas the macrophotographs of parts of the seedlings and the leaf discs have been taken under Semi 2000-C stereomicroscope (Zeiss) equipped with an AxioCam ERc5s (Zeiss) camera and AxioVision Rel. 4.8 (Zeiss) software.

To measure necrotic areas in leaf discs and to estimate cell viability, the ImageJ (2.1.4.7; Tomasz Szydłowski, Poland) and ScnImage (Scion Corporation) software (open source) were applied, respectively.

To freeze dry plant samples and evaporate treatment solutions, a rotatory evaporator (CenriVap, Labconco) was used. HPLC analyses of BPA levels in plant samples were performed using Dionex Ultimate 3000 equipped with a LiChroCart C-18 (250 × 3 mm, 5 µm, Merck) column, UV–Vis detector and autosampler controlled digitally with Chromeleon 6.80 software.

All spectrophotometric measurements (amount of phenols, ACC, quinones, sugars and proteins as well as BPA) were performed using an Ultrospec 110 pro Amersham spectrophotometer.

To assess the impact of BPA, respective Pearson coefficients (***r***_***xy***_; Microsoft Excel) showing correlations (i) between BPA increasing concentrations versus a chosen studied trait as well as (ii) between two different traits (x and y) were determined (Fig. 9A–D). This coefficient varies between + (plus)1 and – (minus)1 depending on the direction of the correlation. Values of |***r***_***xy***_| between 0.0 and 0.4, between 0.41 and 0.8 and greater than 0.81 were considered to indicate no, moderate, and strong correlations, respectively. Values greater than 0.40 were analysed as statistically significant at *p* ≤ 0.05.

Picosmos Tools (open source), CORELDRAW GRAPHICS SITE X7 EDULIC (licenced) and/or INSCAPE (open source; https://inkscape.org) were used to prepare figures and image planes in png extensions. Images of seedlings were taken using a Canon 100 (Japan) private camera in jpg extension.

### Ethics approval

Authors confirm that all experiments with seedlings of not genetically modified plant of *Vicia faba* ssp. minor were performed in accordance with relevant guidelines and regulations using operation instruction of the laboratory equipment and measuring instruments.

All applied to study methods were performed in accordance with the relevant guidelines and regulations using the protection equipment against hazards.

Moreover, all images of the manuscript of the paper are the effects of the authors collaborations.

Authors confirmed that permission of usage of seeds of *V. faba* spp. minor var. ‘Bobas’ for scientific application in the Department of Cytophysiology, Pomorska 141/143, 90-236 Łódź, Poland was obtained by Danko Hodowla Nasion Sp. z o.o. (http://www.danko.pl).

### Statement of compliance

Experiments on plants in the present study were performed with international, national and/or institutional guidelines. It means that the number of seeds used to germination was depended on seed germination rates and it was directly related with the numbers of seedlings planed to used during experiments.

## Supplementary Information

Supplementary Information.

## Data Availability

The datasets generated and analyzed during the current study are available from the corresponding author on reasonable request.
