# Supplementary material for: Influence of bisphenol A on growth and metabolism of Vicia faba ssp. minor seedlings depending on lighting conditions
Source: Sci Rep. 2022 Nov 24;12:20259. doi: 10.1038/s41598-022-24219-0 (PMC9691730; doi:10.1038/s41598-022-24219-0)
Supplement: Supplementary file 1 — Supplementary Information. [file 41598_2022_24219_MOESM1_ESM.pdf]

Influence of bisphenol A on growth and metabolism of *Vicia faba* ssp. minor seedlings depending on lighting conditions

Kaźmierczak Andrzej [1]✉, Kornaś Andrzej [2], Mościpan Małgorzata [3], Justyna Łęcka [4]

[1] Andrzej Kaźmierczak (A.Ka.)
University of Łódź, Faculty of Biology and Environmental Protection, Institute of Experimental Biology, Department of Cytophysiology, Pomorska 141/143, 90-236 Łódź, Poland

[2] Andrzej Kornaś (A.Ko.)
Institute of Biology, Pedagogical University of Krakow, Podchorążych 2, 30-084 Kraków, Poland
andrzej.kornas@up.krakow.pl
https://orcid.org/0000-0001-9945-3322

[3] Małgorzata Mościpan (M.Mo.)
Institute of Heavy Organic Synthesis "Blachownia", Energetyków 9, 47-225 Kędzierzyn-Koźle, Poland
malgorzata.moscipan@poczta.pl

[4] Justyna Łęcka (J.Ł.)
Department of Inorganic and Analytical Chemistry, Faculty of Chemistry, Laboratory of Environmental Threats, University of Lodz, Tamka 12, 91-403 Lodz, Poland
justyna.lecka2@edu.uni.lodz.pl

(✉Corresponding author)

E-mail address: andrzej.kazmierczak@biol.uni.lodz.pl

Fax: +48 +42 635-45-12

Phone: +48 +42 635-42-03

ORCID: 0000-0001-6446-7337; ID: 56261384600

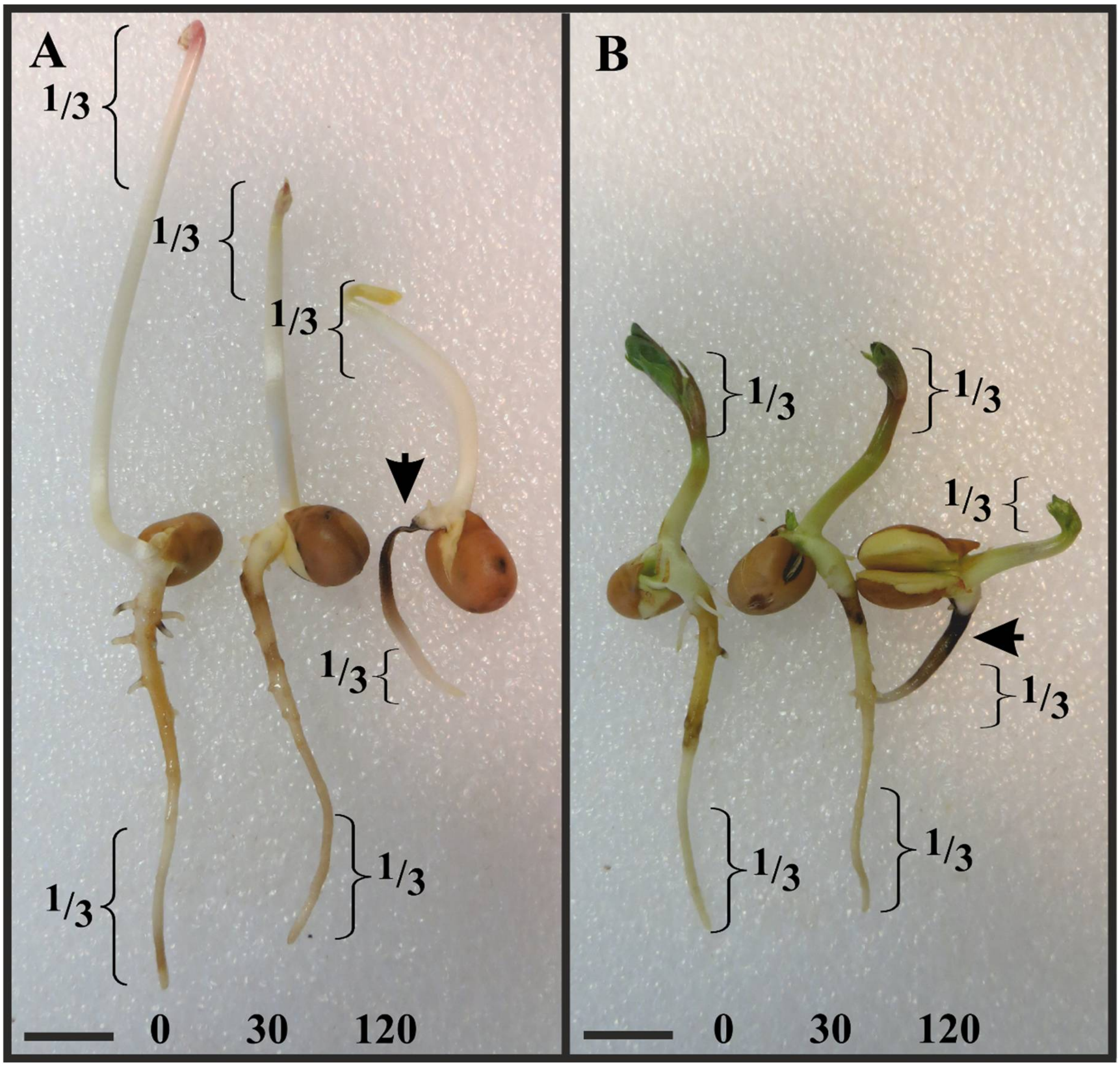

Figure S1. *V. faba* ssp. *minor* seedlings cultured in the darkness - DK (a) and in the darkness/light – DK/LT (b) under Ctrl (control; 0 mg BPA) conditions or with 30 or 120 mg L^{-1} BPA for 72 h. Clamps indicate one-third (1/3) of the apical parts of stems and roots that were used for metabolic analyses. Arrows point to the necrotic areas in roots. Scale bar = 1 cm.

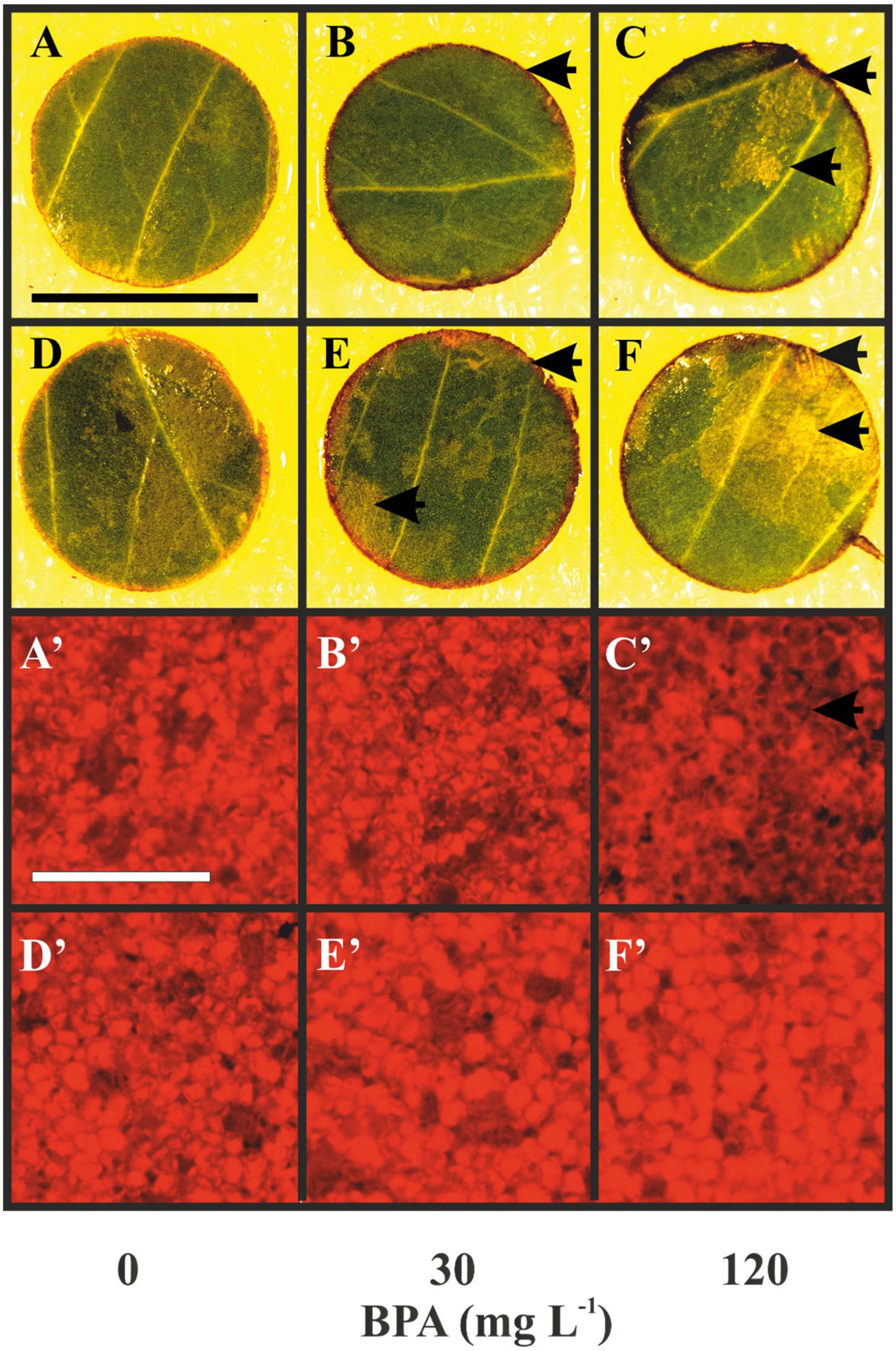

Figure S2. Images of leaf discs (1-cm) of 2-month-old *V. faba* ssp. *minor* plants observed under a stereoscopic microscope (A–F) and fragments of these discs under fluorescence blue light (A'–F'). The discs were cultured for 72 h in darkness (DK; A–C; A'–C') and darkness/light (DK/LT; D–F; D'–F) under Ctrl (0 mg BPA) conditions (A,D) or with 30 (B,C) and 120 mg L^{-1} (C,F) BPA. In "A", the scale bar = 1 mm and is applied to B–F. In "A', the scale bar =100 μm and is applied to B'–F'. Arrows point to necrotic areas.

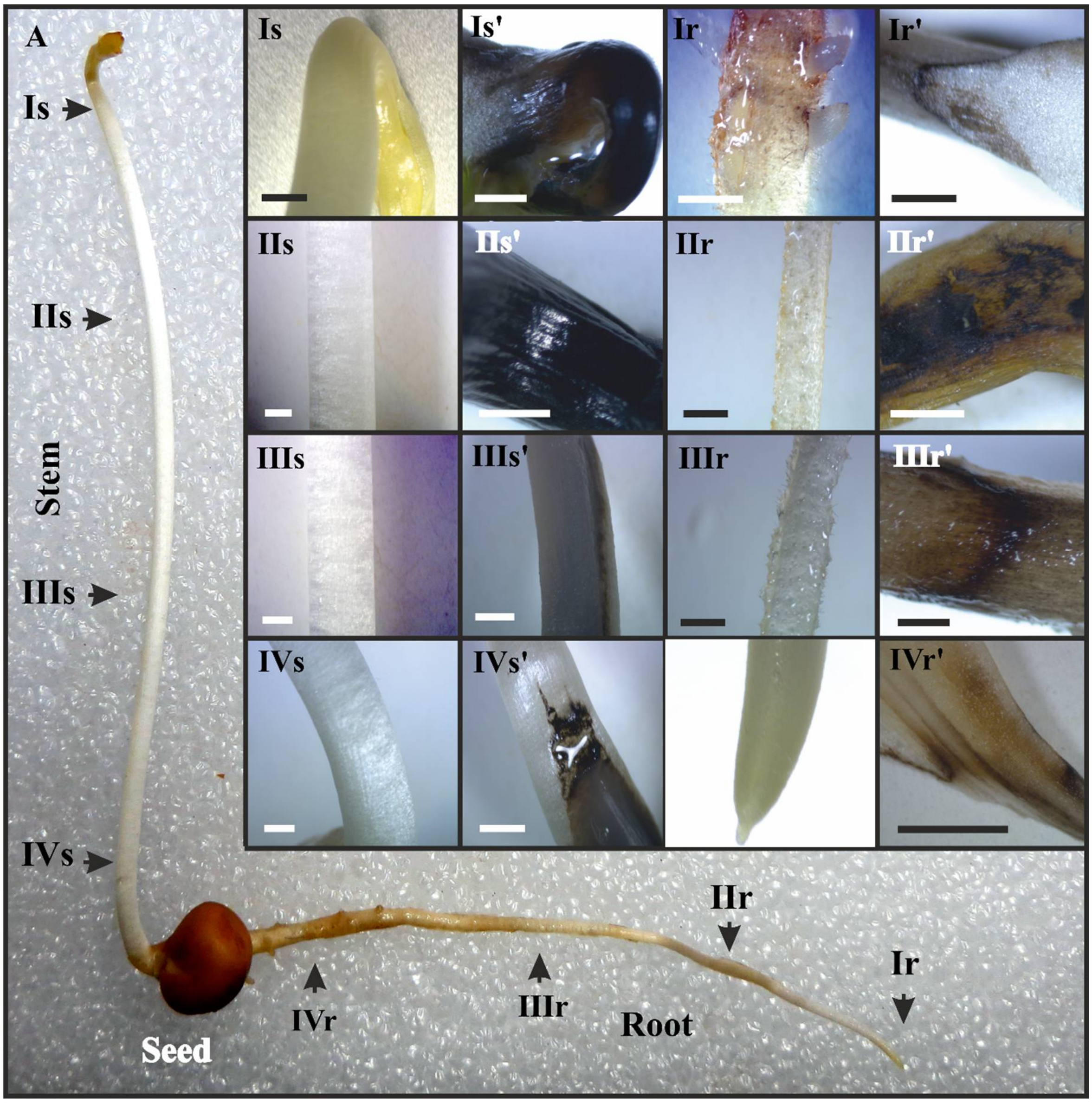

Figure S3. Ctrl (0 mg BPA) *V. faba* ssp. *minor* seedling and its fragments indicated by arrowheads: apical (Is, Ir), subapical (IIs – stem, IIr – root), subbasal (IIIs, IIIr) and basal (IIIs, IIIIr) parts of stem (A–D) and root (E–H) in which necrosis (A'–D' and E'–H', respectively) was observed after 72 h treatment with 120 mg L-1 BPA in the darkness (DK).

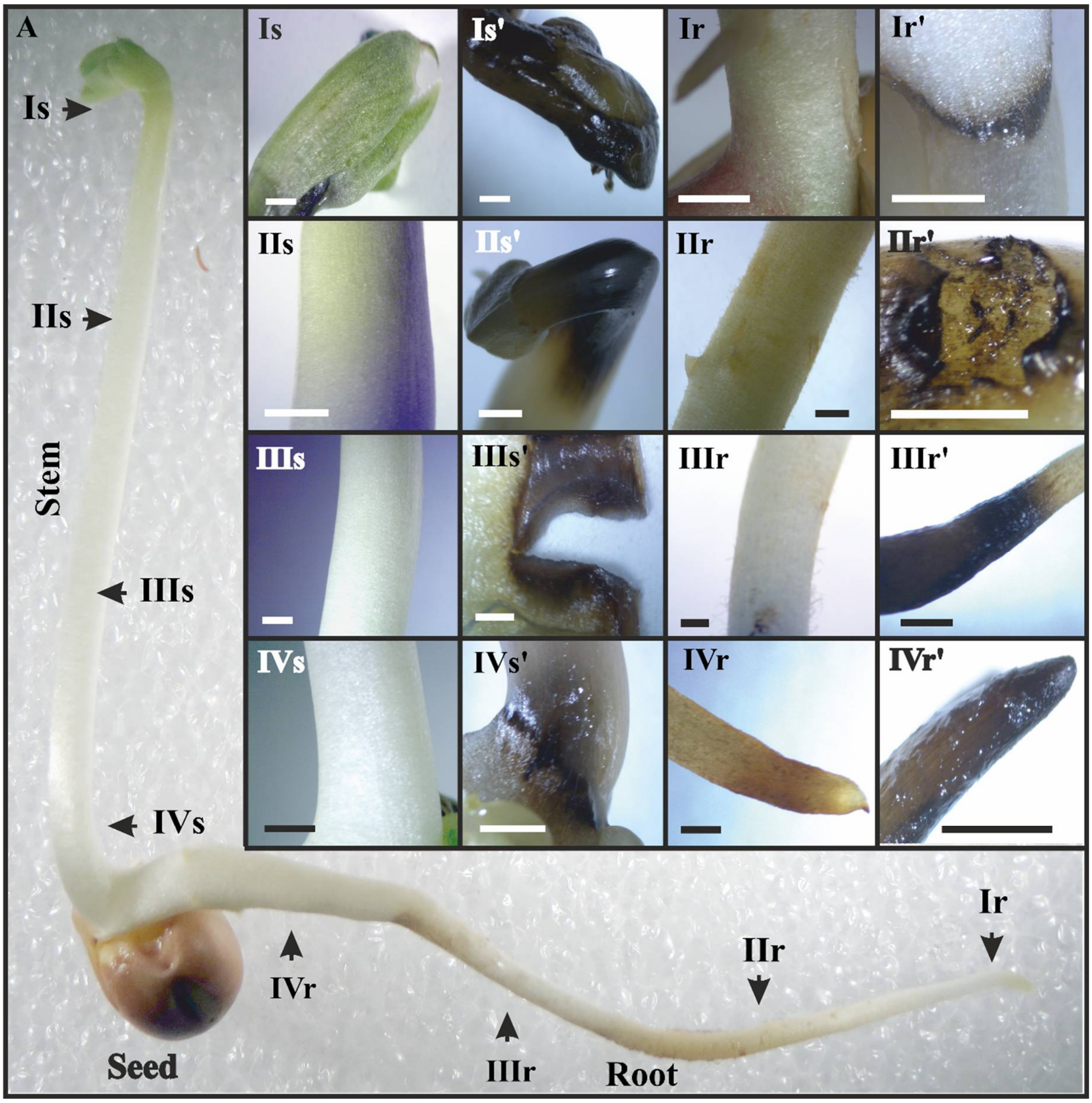

Figure S4. Ctrl (0 mg BPA) *V. faba* ssp. *minor* seedling and its fragments indicated by arrowheads: apical (Is, Ir), subapical (IIs - stem, IIr - root), subbasal (IIIs, IIIr) and basal (IIIs, IIIIr) part of stem (A–D) and root (E–H) in which necrosis (A'–D' and E'–H') respectively was observed after 72 h treatment with 120 mg L^{-1} BPA in the darkness/light (DK/LT). Scale bars = 1 mm

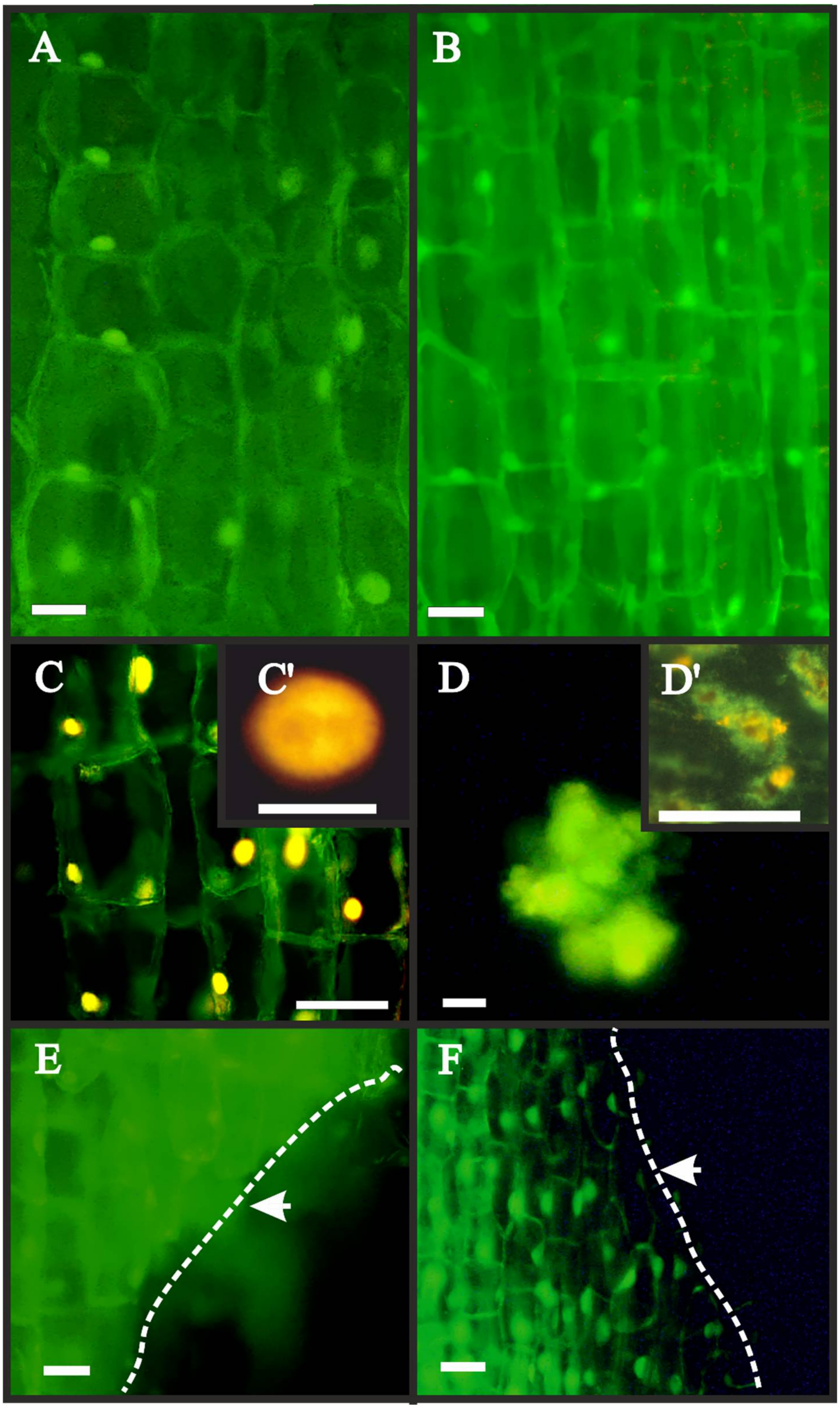

Figure S5. Fluorescent microphotographs of the parts of roots (A) and stems (B). Here, green nuclei indicate living cells (A,B), and red nuclei indicate dead cells (C,C'). There are also totally degraded nuclei (D,D'). In addition, parts of roots (E) and stems (F) are noted with a border (white line) observed between living and dead (without nuclei) parts of *V. faba* ssp. *minor* seedlings treated with 120 mg L^{-1} BPA for 72 h. Arrows point to dead parts of roots and stems. Scale bars = 20 μm.

List	Description
Keywords	bisphenol A; cell death/viability, chlorophyll; ethylene, fluorescence microscopy; hydrogen peroxide, necrosis; phenols, photosymthesis; quinones
Abbreviations	abscisic acid (ABA); acridine orange (AO); bisphenol A, (BPA); cell wall-bound sugars, (CWS); chlorophyll *a*, (chl *a*); darkness (DK); darkness/light (DK/LT); ethidium bromide (EB); ethylene (ETH); empiric parameter used to assess plant vitality, (R_{fd}); fluorescence intensity (RFI); indole-3-acetic acid (IAA); intensity of red fluorescence of chlls (FICh); gibberellic acid (GA); maximum fluorescence yield, quantum yield in darkness/light -adapted steady-state, (NPQ); peroxide, (PX); photochemical quenching, (qP); photosystem II, (PSII); programmed cell death, (PCD); ratio of variable to maximum fluorescence – the quantum efficiency of open PSII centers, (F_v/F_m); soluble sugars, (SOS); steady-state PSII quantum yield in DK/LT (QY); storage sugars (STS); zeatin (ZT).
